# Oligomer-targeting with a conformational antibody fragment promotes toxicity in Aβ-expressing flies

**DOI:** 10.1186/2051-5960-2-43

**Published:** 2014-04-11

**Authors:** Jessica Wacker, Raik Rönicke, Martin Westermann, Melanie Wulff, Klaus G Reymann, Christopher M Dobson, Uwe Horn, Damian C Crowther, Leila M Luheshi, Marcus Fändrich

**Affiliations:** 1 Max-Planck Research Unit for Enzymology of Protein Folding, 06120 Halle, Saale, Germany; 2 German Centre for Neurodegenerative Diseases (DZNE), 39118 Magdeburg, Germany; 3 Electron Microscopy Center, Jena University Hospital, Friedrich Schiller University Jena, 07743 Jena, Germany; 4 Institute for Pharmaceutical Biotechnology, Ulm University, Helmholtzstr. 8/1, 89081 Ulm, Germany; 5 Department of Chemistry, University of Cambridge, Cambridge CB2 1EW, UK; 6 Leibniz Institut für Naturstoff-Forschung und Infektionsbiologie, Hans-Knöll-Institut, 07745 Jena, Germany; 7 Department of Genetics, University of Cambridge, Cambridge CB2 3EH, UK; 8 Cambridge Institute for Medical Research, Cambridge CB2 0XY, UK; 9 Present address: MVZ Labor Dessau GmbH, Bauhüttenstr. 6, 06847 Dessau, Germany

**Keywords:** Conformational disease, Misfolding, Neurodegeneration, Prion, Protein aggregation

## Abstract

**Introduction:**

The self-assembly of Aβ peptides into a range of conformationally heterogeneous amyloid states represents a fundamental event in Alzheimer’s disease. Within these structures oligomeric intermediates are considered to be particularly pathogenic. To test this hypothesis we have used a conformational targeting approach where particular conformational states, such as oligomers or fibrils, are recognized *in vivo* by state-specific antibody fragments.

**Results:**

We show that oligomer targeting with the KW1 antibody fragment, but not fibril targeting with the B10 antibody fragment, affects toxicity in Aβ-expressing *Drosophila melanogaster*. The effect of KW1 is observed to occur selectively with flies expressing Aβ(1–40) and not with those expressing Aβ(1–42) or the arctic variant of Aβ(1–42) This finding is consistent with the binding preference of KW1 for Aβ(1–40) oligomers that has been established *in vitro*. Strikingly, and in contrast to the previously demonstrated *in vitro* ability of this antibody fragment to block oligomeric toxicity in long-term potentiation measurements, KW1 promotes toxicity in the flies rather than preventing it. This result shows the crucial importance of the environment in determining the influence of antibody binding on the nature and consequences of the protein misfolding and aggregation.

**Conclusions:**

While our data support to the pathological relevance of oligomers, they highlight the issues to be addressed when developing inhibitory strategies that aim to neutralize these states by means of antagonistic binding agents.

## Introduction

Amyloid fibrils are filamentous polypeptide aggregates characterizing a range of human diseases, including systemic amyloidosis and a variety of neurodegenerative conditions [1-4]. In Alzheimer’s disease they are formed from β-amyloid (Aβ) peptide [5-8]. This peptide is able to aggregate into a multitude of different assembly states [2-4]. It also occurs in different chemical isoforms [9-11], but oligomeric intermediates of fibril formation are often reported to play a pivotal role in AD pathogenesis [6-8,12,13]. Much of the evidence supporting this view has come from *in vitro* toxicity measurements or structures that were prepared inside the test tube or extracted from its native sources by highly invasive biochemical methods [6,8,12,14-16]. Oligomers have thus been suggested as targets of therapeutic or inhibitory strategies ameliorating AD, and oligomer binding ligands, including conformational antibodies, were shown to antagonize their anti-neuronal activity in various assay systems [17-21].

To probe for the presence and relevance of specific Aβ states *in vivo*, we have carried out a conformational targeting study, introducing two biophysically well-characterised conformation-specific Aβ antibody fragments into a *Drosophila* model of Aβ toxicity. Fly models are well defined model systems and gave important insights into the toxicity of Aβ and other polypeptide chains [22-28]. For our study we used flies expressing the 40 and 42 amino acid isoforms Aβ(1-40) and Aβ(1-42) and the disease-associated E22G mutant of Aβ(1–42), termed Aβ(1-42)arc [10] as well as two antibody fragments that were generated previously through a biotechnological approach. That is, they were phage display selected based on their ability to discriminate between different conformational states of Aβ from a fully synthetic library of camelid heavy chain antibody fragments [17,29].

**Figure 1:**
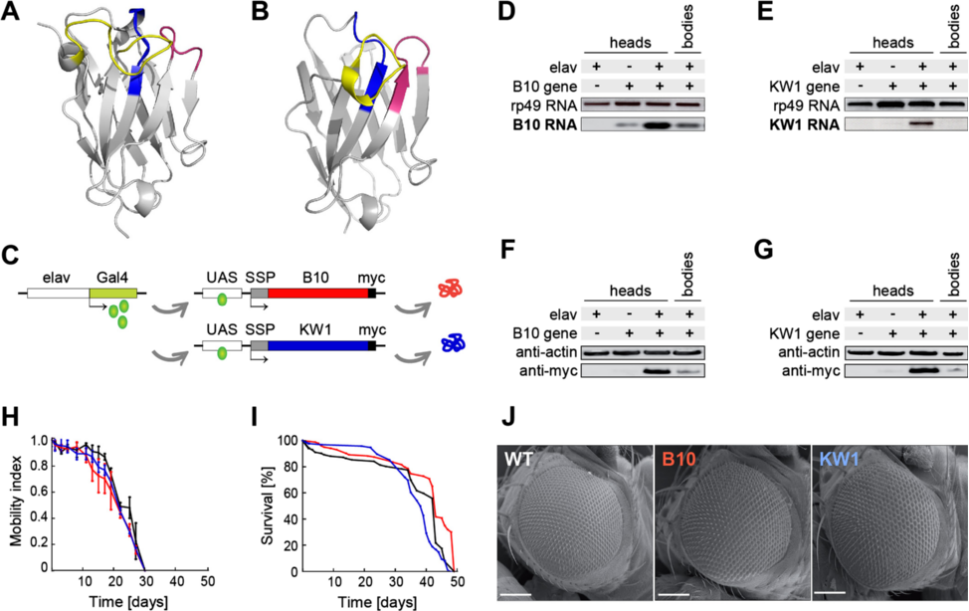
**B10 or KW1 do not noticeably affect the fly phenotype when expressed without Aβ. (A, B)** Ribbon diagrams of the crystal structures of B10 **(A)** and KW1 **(B)**, according to protein data base entries 3LN9 and 3TPK [29,31]. The complementary determining regions (CDRs), that define the antigen binding sites, are coloured in blue (CDR1), red (CDR2) and yellow (CDR3). **(C)** Schematic representation of the expression constructs. The *elav* promoter drives the neuron-specific expression of Gal4 protein, which subsequently induces the neuronal expression of KW1/B10 through binding of an upstream acting sequence (UAS). **(D, E)** Reverse transcription polymerase chain reaction with head and body (thorax and abdomen) homogenates from different fly lines probed for transcription of B10 **(D)** or KW1 mRNA **(E)**. Constitutively transcribed rp49 mRNA serves as a loading control. **(F, G)** WB shows a strong myc-positive band in B10 **(F)** and KW1 flies **(G)** at ~17 kDa molecular weight. Actin staining serves as a loading control. **(H-J)** Phenotypic comparison of B10 (red), KW1 (blue) and WT flies (black). **(H)** Locomotive assay. Error bars present standard deviation from three independent experiments using 15 flies each. **(I)** Viability assay. **(J)** Scanning electron microscopy images of the eyes. Scale bars represent 20 μm.

These monoclonal antibody fragments introduced into the flies are termed KW1 and B10 (Figure 1A,B) [29,30]. KW1 binds oligomers of Aβ(1–40), while fibrils and disaggregated, that is largely monomeric, Aβ(1–40) peptide, or oligomers of the more aggregation-prone Aβ(1-42) peptide are not specifically recognized [14]. Bivalent KW1 binds to Aβ(1–40) oligomers with an apparent dissociation constant (aK_D_) of 43.5 nM and antagonizes oligomer toxicity in long term potentiation (LTP) electrophysiology experiments [14]. B10, by contrast, recognizes Aβ(1–40) fibrils (aK_D_ = 7 nM, determined for bivalent B10), but no oligomers or disaggregated peptide [31,32]. B10 is less selective than KW1 and binds fibrils formed from a broad range of polypeptide chains, including those of Aβ(1–40) and Aβ(1-42) [30-34].

These properties, combined with the ability to promote the formation of either protofibrillar (B10) or non-fibrillar aggregates (KW1) of Aβ [29,30,35], and to bind to their corresponding conformers in human tissue samples [29,30,32], enable B10 and KW1 to be used to probe in a precise and well-defined manner how the binding of particular Aβ conformers in a *Drosophila* model of Aβ toxicity affects its self-assembly and consequent neurodegeneration *in vivo*. By correlating these observations with parallel studies of the effects of these antibody fragments on the formation of cytotoxic aggregates *in vitro*, we are able to rationalise their ability (or lack of ability) to modulate the neurotoxicity of each Aβ isoform *in vivo* on the basis of their conformational selectivity.

## Materials and methods

### Preparation of different Aβ conformers

The Aβ(1–40) peptide was recombinantly expressed in house [36], while the Aβ(1–42) and Aβ(1–42)arc peptides were obtained from chemical synthesis (Dr. Sven Rothemund, University Leipzig, Germany). The purity was > 96%, based on reverse phase high performance liquid chromatography. Fibrils were formed *in vitro* by incubation of pure peptide at 1 mg/ml concentration in 50 mM sodium borate buffer, pH 9.0, for 5 days at room temperature. Oligomers were prepared by dissolving pure peptide at 2.5 mg/ml concentration in 100% 1,1,1,3,3,3-hexafluoro-2-propanol [37]. After incubation for 15 min at room temperature, the solution was diluted 10-fold with H_2_O and further incubated for 15 min. Large aggregates were then removed by spinning down the sample at 14,000 × *g* for 15 min. The supernatant containing the oligomers was used for further analysis.

### Generation of B10 or KW1 transgenic flies

The coding sequences for KW1 and B10 were obtained by chemical synthesis (GeneArt), codon optimized for *Drosophila melanogaster*. The genes were cloned into the Gal4-responsive pUAST/attB expression vector (kind gift of Konrad Basler; [38] and flanked by a 5′-SSP sequence from *Drosophila necrotic* gene [39] and a 3′-myc-tag (Additional file 1: Figure S1A). B10 and KW1 transgenic flies were commercially generated by PhiC31 integrase-mediated transgenesis using the attP landing line zh-51D (Best Gene Inc.). Through polymerase chain reaction of the genomic DNA from the two initial fly lines +/+;B10/CyO;+/+and +/+;KW1/CyO;+/+we confirmed the presence of the KW1/B10 constructs within the genomic DNA. B10 or KW1-expressing flies were obtained through further crossing of these flies with the Gal4-elav^c155^ pan-neuronal driver strain (Bloomington). The Gal4-UAS system involves an upstream activating sequence (UAS) element that activates the transcription of KW1 or B10 when bound by Gal4 protein [40]. Gal4 expression, in turn, is controlled by the pan-neuronal *elav*^*c155*^ promoter, which induces a neuron-specific expression pattern of Gal4 protein along with KW1 or B10. The resulting strains are referred to in the text as B10 or KW1 flies.

To co-express B10 or KW1 with Aβ, we crossed the commercially obtained +/+;B10/CyO;+/+and +/+;KW1/CyO;+/+lines with flies transgenic for Aβ(1–40), Aβ(1–42) or Aβ(1–42)arc that had not previously been crossed with the Gal4-elav^c155^ driver strain. This initial cross resulted in the three initial B10 and Aβ double-transgenic strains +/+;B10/CyO;Aβ(1–40)/TM6B, +/+;B10/CyO;Aβ(1–42)/TM6B and +/+;B10/CyO;Aβ(1–42)arc/TM6B as well as the three KW1 and Aβ double-transgenic strains +/+;KW1/CyO;Aβ(1–40)/TM6B, +/+;KW1/CyO;Aβ(1–42)/TM6B and +/+;KW1/CyO;Aβ(1–42)arc/TM6B. Through a second cross of these flies with the Gal4-elav^c155^ strain, we induced the expression of the transgenic proteins. The resulting fly lines are referred to within the main text as B10;Aβ40, B10;Aβ42, B10;Aβ42arc, KW1;Aβ40, KW1;Aβ42 and KW1;Aβ42arc flies. They neuronally express the *gal4* gene, which encodes the yeast transcription activator Gal4. Gal4 protein binds to the UAS element, which activates the neuron-specific transcription of Aβ, KW1 or B10.

In addition, we generated two further fly lines that were co-expressing Aβ(1–40) and KW1. These lines were obtained by crossing +/+;KW1/CyO;+/+flies with the Gal4-elav^c155^ strain to obtain elav/elav;KW1/CyO;+/+flies. The resulting animals were then crossed with the Aβ(1–40) peptide expressing lines Aβ(1–40)-29.1, which behaves identically to Aβ40 flies [22], and Aβ(1–40)-51D [41] flies to generate the lines KW1;Aβ40-29.1 and KW1;Aβ40-51D. The respective control lines (no KW1 expression) were obtained by crossing Aβ(1–40)-29.1 and Aβ(1–40)-51D flies with the Gal4-elav^c155^ strain to generate the Aβ(1–40) expressing lines Aβ40-29.1 and Aβ40-51D. All crosses were carried out at 25°C on standard *Drosophila* food with dried yeast [42].

### Survival assay

Ten groups of ten flies, were incubated at 29°C in 4-inch glass vials (100 flies in total). The animals were kept on standard food with dried yeast, and the food was replenished every 2 days. The number of living flies was counted every 2–3 days, and the resulting survival curves were evaluated with the Kaplan-Meier method that is implemented within the Sigma Plot11 software (Systat Software Inc). Differences between the genotypes were assessed by using the log-rank test (Sigma Plot11). All survival times quoted in the text and in Table 1 represent median values ± standard error of mean.

**Table 1:** Median survival time of the fly lines

**Expressed Aβ variant**	**Expressed antibody fragment**		
	**None**	**B10**	**KW1**
None	43 ± 0.1	43 ± 0.2	42 ± 1.1
Aβ(1–42)arc	7 ± 0.1	6 ± 0.3	7 ± 0.2
Aβ(1–42)	32 ± 0.3	31 ± 0.6	32 ± 0.3
Aβ(1–40) (line Aβ40)	43 ± 0.6	41 ± 0.9	28 ± 1.1
Aβ(1–40) (line Aβ40-51D)	42 ± 1.1	-	36 ± 0.8
Aβ(1–40) (line Aβ40-29.1)	39 ± 0.8	-	39 ± 0.4

**Figure 2:**
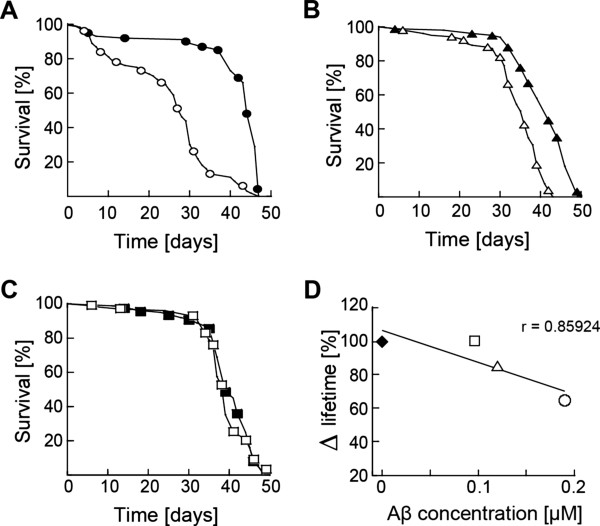
**KW1 expression reduces the viability of different Aβ(1–40) expressing fly lines. (A-C)** Lifespan measurements of Aβ40 flies (filled) and KW1;Aβ40 flies (unfilled) **(A)**, Aβ40-51D flies (filled) and KW1;Aβ40-51D flies (unfilled) **(B)** and Aβ40-29.1 flies (filled) and KW1;Aβ40-29.1 flies (unfilled) **(C)**. **(D)** Fly viability versus Aβ concentration in the fly head. The fly viability (%) is given relative to fly lines without KW1 expression. Symbols: Filled diamond: KW1 relative to WT. Unfilled square: KW1;Aβ40-29.1 relative to Ab40-29.1. Unfilled triangle: KW1;Aβ40-51D relative to Aβ40-51D. Unfilled circle: KW1;Aβ40 relative to Aβ40. Data were fitted by linear regression (R = 0.86). For original values please refer to Table 1.

Values are given in days. Original data are shown in Figures 1 and 2 and Additional file 1: Figure S4. Errors represent standard error of mean.

### Negative geotaxis assay

A negative-geotaxis assay was used to determine the locomotor ability of the flies. A total of 15 flies (*n*_total_) were placed in a 25 ml plastic pipette and knocked to the bottom. After 45 seconds, the number of flies that had reaching the top of the pipette (*n*_top_), as defined by the 25 ml-mark, was determined. The number of flies remaining at the bottom (*n*_bottom_) was defined by the 2 ml-mark. From these numbers the mobility index was calculated according to the equation (*n*_total_ + *n*_top_ − *n*_bottom_)/2 ⋅ *n*_total_. This measurement was repeated three times with independent groups of flies, consisting of 15 animals each. Climbing index values quoted in the text represent mean values from the three independent measurements ± standard error of mean.

### Scanning electron microscopy analysis of the eye morphology

Ten 1-day old adult animals were collected from each analyzed fly line, anesthetized with carbon dioxide and fixed over night in 500 μl of a 100 mM sodium cacodylate solution (pH 7.3) containing 2.5% glutaraldehyde (4°C). The fixative was removed by washing the flies 5 times for 5 min in 500 μl sodium cacodylate buffer without glutaraldehyde, followed by a series of 5 dehydration steps (500 μl each), where the ethanol concentration was progressively raised from 10 to 100%. The organic solvent was removed by critical point drying in a BAL-TEC CPD 030 Critical Point Dryer. All samples were evacuated and sputtered with gold (layer thickness 20 nm) in a BAL-TEC SCD 005 Sputter Coater. The final analysis was done using a scanning electron microscope Zeiss (LEO) 1450 VP at 8 kV acceleration voltage and pictures were taken at 200 × magnification.

### Immunohistochemistry

Whole brains were dissected from adult fly heads and transferred into 500 μl ice cold PBS. For fixation the brains were then transferred into 4% (w/v) paraformaldehyde in PBS for 1 h at room temperature. Afterwards the fixative was removed and the brains were blocked with 5% normal goat serum in PBS (15 min). The brains were then infiltrated for 2 h with 6E10 (Aβ1-16, monoclonal, mouse, Covance, 1:500), anti-elav (monoclonal, rat, DHSB, 1:50) or anti-myc antibody (ab9106, polyclonal, rabbit, Abcam, 1:500). All antibodies were diluted in PBT (PBS + 0.05% TritonX-100). Unbound antibodies were removed with three washing steps (500 μl PBT, 10 min each) followed by a 1 h incubation with donkey anti-mouse IgG Alexa Fluor®555, donkey anti-rabbit IgG Alexa Fluor®488 (Molecular Probes, 1:200) or anti-rat IgG TRITC (JacksonImmuno Research, 1:200) as appropriate. To remove unbound secondary antibodies, the brains were washed three times with 500 μl PBT, before they were incubated 10 min with 500 μl Hoechst 33342 dye (1 μg/ml in PBS) to stain the nuclei. Finally, the brains were mounted using VECTASHIELD® mounting medium (Vector laboratory). All steps were performed at room temperature, unless indicated otherwise. All samples were analysed using a Nikon ECLIPSE TE2000-E confocal laser-scanning microscope. Pictures were taken at a magnification of 60 ×.

### Protein extraction from flies

Flies were anesthetized, shock frozen on dry ice and decapitated. Depending on whether the proteins samples were supposed to be used for denaturing sodium dodecylsulphate (SDS) polyacrylamide gel electrophoresis (PAGE) or native PAGE we then used slightly different extraction methods. Denaturing SDS PAGE analysis: we homogenized 20 fly heads or 5 fly bodies in 20 μl phosphate-buffered saline (PBS, 137 mM NaCl; 2.7 mM KCl; 8 mM Na_2_HPO_4_; 2 mM KH_2_PO_4_; pH 7.4), which was supplemented with 1% SDS and the complete protease inhibitor cocktail (Roche Applied Science). Homogenization was performed manually by using a plastic pistil. The homogenates were sonicated for 8 min and spun down for 7 sec, before the supernatants were collected. The protein concentrations in these samples were determined with the DC Protein Assay (Biorad), and the protein concentration was adjusted in the various samples to ensure equal loading on the gel. All samples were then mixed with 5 μl 4× NuPAGE® sample buffer (Invitrogen) and heated for 10 min at 95°C. The samples were then separated on NuPAGE® 4–12% Bis-Tris gradient gels with NuPAGE® MES SDS running buffer (Invitrogen).

For native PAGE analysis, we manually homogenized 20 fly heads in 20 μl PBS, supplemented with the cOmplete protease inhibitor mix. This suspension was sonicated for 8 min, spun down for 7 sec to remove insoluble material, and the supernatant was mixed with 5 μl of 4× NativePAGE® sample buffer (Invitrogen). These electrophoresis samples were not boiled before they were separated on NativePAGE® 4-16% Bis-Tris gradient gels with NativePAGE® running buffer (Invitrogen). The further readout of the results was performed with WB analysis, if required.

### RNA extraction and polymerase chain reaction

To check the transcription of the KW1 and B10 genes in the animals, we used a three step protocol: RNA extraction from the flies, conversion of RNA into cDNA and analysis of the cDNA to confirm the coding sequences of B10 or KW1.

RNA isolation was based on TRIzol® reagent (Invitrogen). In brief, flies were anesthetized and shock frozen on dry ice in Eppendorf tubes. Flies were decapitated by shaking the Eppendorf tube on dry ice to break the neck of the flies. The heads were manually separated from the remaining bodies. 15–20 heads or 5 bodies were homogenized in 150 μl TRIzol® by using a plastic pistil. After addition of 30 μl chloroform, the mixture was thoroughly vortexed and the organic and aqueous phases were separated by centrifugation (13,000 × *g*, 15 min, 4°C). The aqueous phase was transferred into a new tube and all nucleic acids were precipitated by using 75 μl pure isopropanol, followed by centrifugation at 13,000 × *g* for 15 min at 4°C. The pellet was briefly washed with 150 μl of 70% ethanol, air dried and re-dissolved in 20 μl RNAse free water.

As a next step, 1 μg of the purified RNA was transferred into a fresh tube and the residual genomic DNA was removed by enzymatic digestion with 1 unit DNAse for 30 min at room temperature. All of the resulting pure RNA was then used for reverse transcription polymerase chain reaction to generate cDNA (RevertAid™ First Strand cDNA Synthesis Kit, Fermentas), which was used according to the manufacturer’s instructions.

Finally, 1 μl of the resulting cDNA were subjected to polymerase chain reaction, which consisted of an initial denaturation step [5 min at 95°C] followed by 28 cycles each of which consisted of 30 sec at 95°C [denaturation], 30 sec at 57°C [primer annealing] and 30 sec at 72°C [elongation]. At the end of these cycles we applied a final extension step of 5 min at 72°C. The polymerase chain reaction products were separated on an agarose gel and photographically imaged. Primers used to amplify B10 and KW1 cDNA were designed based on the coding sequences of KW1 and B10 genes. Primers used to amplify Aβ cDNA were designed based on the coding sequences of Aβ transgene. Amplified rp49 cDNA served as a loading control. See Additional file 1: Table S1 for primers.

### Immunoprecipitation

Magnetic protein A coupled beads (Invitrogen) were pretreated by blocking 10 μl of the resuspended beads in 100 μl 2% BSA in PBS + 0.025% TritonX-100. After incubation for 15 min, the beads were washed twice in 100 μl PBS + 0.025% TritonX-100 for 5 min. 3 μg of the dissolved antibody (6E10) were added to 100 μl PBS +0.025% TritonX-100 and incubated with the beads for 15 min to allow coupling of the antibodies to the beads. After this pre-treatment, 10 to 20 fly heads were homogenized with a plastic pistil in 20 μl PBST containing the cOmplete protease inhibitor mix. The suspension was sonicated for 1 min, centrifuged for 7 sec and the supernatant was transferred into a fresh tube. This solution was then diluted with 80 μl PBS + 0.025% TritonX-100, mixed with the pre- treated beads and incubated for 20 min. The beads were washed thrice with 100 μl PBS + 0.025% TritonX-100 (5 min) and transferred into a fresh tube. Specifically bound proteins were eluted from the beads with 20 μl 50 mM glycine (pH 2.8) and incubation for 10 min. Afterwards the beads were boiled in 20 μl 1× NuPAGE® LDS Sample Buffer (Invitrogen) and applied onto the gel to check for unspecifically bound proteins. All steps were performed at room temperature, unless indicated otherwise. The resulting fractions were analysed using WB. Anti-myc antibody was used to detect the expressed myc-tagged B10 antibody fragment, while 6E10 was used against Aβ.

To test for possible interactions between KW1 and Aβ peptide, a modified protocol had to be used, because fly KW1 (unlike fly B10) was found to directly bind to protein A beads. Therefore, the bead pre-treatment consisted only of the blocking of 10 μl resuspended beads (in 100 μl 2% BSA, PBS, 0.025% TritonX-100), incubation for 15 min and two washing steps as described above. After this pre-treatment, 10 to 20 fly heads were prepared as described above and mixed with the pre-treated beads. The further steps were carried out as described above. Anti-myc antibody was used to detect the expressed myc-tagged KW1 antibody fragment via WB, while 6E10 was used against Aβ. All steps were carried out at room temperature.

### Fluorescence spectroscopy

Thioflavin T (ThT) and 8-naphthalene-1-sulfonate (ANS) spectra were recorded at room temperature using the LS 55 fluorescence spectrometer (Perkin Elmer). All spectra represent averages of 5 scans. ThT fluorescence was excited at 450 nm and the emission spectrum was recorded between 460 and 700 nm. All ThT spectra were recorded with samples containing 15 μM ThT and 20 μM Aβ. ANS emission spectra were measured from 380 to 700 nm, whilst exciting at 374 nm. In this measurement samples contained 200 mM ANS and 20 μM Aβ peptide.

### Spot blot

1–20 μg from each Aβ peptide species were blotted onto nitrocellulose membrane (GE Healthcare or Schleicher and Schuell) using pore sizes of 0.1 μm or 0.45 μm. The membrane was blocked for 1 h with 2% bovine serum albumin (BSA) solution in TBST, which is Tris- buffered saline (50 mM Tris, 200 mM NaCl, pH 7.4), containing 0.01% Tween 20. Equal loading was confirmed with Ponceau red staining of unblocked control membranes. Membranes were washed thrice in TBST for 5 min, before they were further incubated for 1 h with 4 μg/ml B10AP or KW1AP in TBST. B10AP or KW1AP binding was visualized with NBT/BCIP reagent (Pierce). All steps were carried out at room temperature. Densitometric quantifications of the scanned blots were carried out with TotalLab 100 software.

### Congo red (CR) absorption spectroscopy

CR absorption was measured at room temperature using the Lambda 900 spectrometer (Perkin Elmer). The samples contained 15 μM CR with or without 25 μM Aβ peptide. Absorbance spectra were recorded from 400 to 700 nm with 3 scans per spectrum. CR absorption spectra of Aβ fibrils or buffer control always contained 50 mM sodium borate buffer, pH 9.0, while Aβ oligomers or controls were measured in 10% 1,1,1,3,3,3-hexafluoro-2-propanol (HFIP).

### Aggregation kinetics measurements

Aggregation kinetic measurments are based on time-resolved ThT fluorescence measurements, carried out online in a 96-well plate and by using a FLUOstar OPTIMA (BMG Labtech) plate reader (37°C). ThT fluorescence was recorded by using excitation and emission wavelengths of 482 nm and 490 nm, respectively. Each measurement cycle consisted of 30 min incubation followed by orbital shaking at 100 rpm for 10 seconds immediately before the measurement. Samples were prepared by initially disaggregating Aβ(1–40) peptide as described [43]. The disaggregated peptide was dissolved at high concentration in 100% dimethyl sulphoxide (DMSO), and the accurate Aβ concentration was determined by absorbance spectroscopy [44]. To that end we diluted 10 μl of the DMSO-stock with 190 μl pure water and 800 μl of 7.5 M guanidine hydrochloride, 25 mM sodium phosphate buffer, pH 6.5. The optical density at 280 nm was recorded with a Helios γ UV–vis spectrophotometer, and the concentration was derived based on a theoretic molar extinction coefficient of 1280 M^−1^ cm^−1^. From the DMSO stock aliquots were diluted with buffer and other reagents to yield the final sample. These samples contained always a volume of 100 μl, 25 μM Aβ(1–40), 20 μM ThT, 50 mM HEPES buffer (pH 7.4), 50 mM NaCl, a protease inhibitor cocktail (Complete mini, Roche) (1×) and, where appropriate, 5 μM KW1. The DMSO concentration was always less than 5%.

### Transmission electron microscopy (TEM)

TEM specimens of Aβ oligomers and fibrils were prepared by placing 5 μl of each sample solution onto a Formvar/carbon copper grid (200 mesh, Plano) followed by 1 min of incubation. The grid was washed by dipping it subsequently into 3 droplets of water (~50 μl) and the specimen were counterstained with 3 droplets (~50 μl) of 2% (wt/vol) uranyl acetate. Samples were examined using a Zeiss 900 electron microscope that was operated at an acceleration voltage of 80 kV. Samples were imaged at a magnification of 30,000 × .

### Long term potentiation (LTP) measurements

For measuring the influence of various Aβ species on LTP isolated hippocampal slices (400 μm thickness) were prepared from 4-months old C57/B16 mice as described previously [45]. The slices were maintained in a pre-chamber containing 8 ml carbogen-gasified artificial cerebrospinal fluid (ACSF, 124 mM NaCl, 25.6 mM NaHCO_3_, 1.2 mM KH_2_PO_4_, 4.9 mM KCl, 2.5 mM CaCl_2_, 2 mM MgSO_4_, 10 mM glucose).

We prepared three different Aβ-containing samples by incubation of 100 μM Aβ with or without 20 μM KW1 in 50 mM HEPES buffer (pH 7.4) and 50 mM NaCl for 5 days at 37°C without shaking. The sample incubated without KW1 was divided into two parts. One was applied to the slice as it was, to the other one we added KW1 15 min before addition to the slices. Sample solutions containing 1 μM Aβ were applied to the slices in the pre-chamber for 2 hours. The respective control solution contained no Aβ. The slices were then transferred into a submerge-type recording chamber and were allowed to recover for at least 30 min before starting the electrophysiological experiments. The recording chamber was constantly perfused with ACFS at a rate of 2.5 ml/min at 32 ± 1°C.

Synaptic responses were elicited by stimulation of the Schaffer collateral commission fibers in the stratum radiatum (CA1 region) using lacquer-coated stainless steel stimulating electrodes. Glass electrodes (filled with ACSF, 1–4 MΩ) were placed in the apical dendritic layer to record fEPSPs. The initial slope of the fEPSP was used as a measure of this potential. The stimulus strength of the test pulses was adjusted to 30% of the EPSP maximum. During baseline recording, single stimuli were applied every minute (0.0166 Hz) and were averaged every 5 min. Once a stable baseline had been established, long-term potentiation was induced by applying 100 pulses at an interval of 10 ms and a width of the single pulses of 0.2 ms (strong tetanus) three times at 10 min intervals.

### Protein structure representation

KW1 and B10 crystal structures were displayed as ribbon diagrams by using the program PyMOL (DeLano Scientific). The structures have the following protein database identification numbers: 3LN9 (B10) and 3TPK (KW1) [29,31].

### Recombinant expression of B10AP and KW1AP in E.coli

B10AP and KW1AP were recombinantly expressed in *E. coli* and purified according to established procedures [29,30].

### Cultivation of SH-SY5Y and measurements of metabolic/toxic activity

SH-SY5Y cells were grown in Dulbecco’s Modified Eagle Medium (DMEM, PAA Laboratories) supplemented with 10% heat-inactivated fetal bovine serum and 2% Pen/Strep (PAA Laboratories) at 37°C with 10% CO_2_. Cells were seeded into 96-well plates at a density of 50 000 cells/well in 100 μl cell culture medium and grown at 37°C. After 24 h the medium was removed and fresh medium was added together with the Aβ samples or controls to be analyzed.

The Aβ samples were obtained by initially dissolving disaggregated Aβ at high concentration in 100% DMSO. The peptide was then quantified and diluted to a final concentration of 100 μM into 50 mM HEPES buffer pH 7.4, containing 50 mM NaCl. If applicable, the solution also contained 20 μM B10 or KW1, and it was incubated for different periods of time at 37°C. After incubation the Aβ-containing analytes were added to the cells to reach a final concentration of 1 μM Aβ peptide. The cells were then incubated for 24 h at 37°C with the analytes before measuring the cellular effects with one of two assays.

Cell metabolic activity was assessed with a FLUOstar Omega 96-well plate reader (BMG LABTECH) by using the Cell Proliferation Kit I (MTT, Roche) or LDH-Cytotoxicity Assay Kit II (BioVision) according to the manufacturer’s protocol. Statistical analyses were carried out using the paired t-test implemented with SigmaPlot11 (Systat software).

## Results

### Oligomer and fibril targeting differentially affect Aβ induced neurotoxicity in vivo

We transgenically expressed B10 or KW1 in *Drosophila melanogaster* flies within the central nervous system using the *elav*^*c115*^*-*Gal4 driver [22] (Figure 1C). An N-terminal secretion signal peptide (SSP) ensures their insertion into the secretory pathway, whereas an additional C-terminal myc-tag was fused to aid their detection (Additional file 1: Figure S1). Reverse transcription polymerase chain reaction (Figure 1D,E) and anti-myc western blotting (Figure 1F,G) of fly head extracts from *Drosophila* expressing B10 or KW1 alone under the control of the *elav*^*c155*^*Gal4* system confirmed that both proteins were stably expressed in KW1 and B10 flies. The two proteins relatively inert properties, as they do not discernibly affect the fly phenotype when expressed in isolation, as determined by locomotive behaviour (Figure 1H), fly longevity (Figure 1I, Table 1) or eye structure (Figure 1J). Western blot shows only very little interactions between KW1 and B10 and endogenous fly proteins (Additional file 1: Figure S2). Expression of B10 and KW1 protein constructs in *Drosophila* Schneider S2 cells demonstrates that fly cells are able to express both antibody fragments as functional proteins that fully reproduce the conformational selectivity (Additional file 1: Figure S3) that we previously established for *E.coli*-derived proteins [29,30].

Flies were generated in which KW1 was co-expressed together with either of the peptides Aβ(1-40), Aβ(1-42) or the early onset AD-associated E22G mutant Aβ(1–42)arc to investigate KW1’s effects on Aβ neurotoxicity. The resulting fly lines are termed KW1; Aβ40, KW1;Aβ42 and KW1; Aβ42arc. Whilst expression of Aβ(1-40) or KW1 alone do not cause significant neurotoxicity in *Drosophila* (Figure 1H-J, Table 1), co-expression of Aβ(1-40) with KW1 results in a significant 35% reduction in fly lifespan, compared to that of Aβ(1-40) expressing flies (from 43 ± 0.6 days to 28 ± 1.1 days; Table 1). Analysis of the effects of KW1 expression in two other Aβ(1–40) peptide-expressing lines (Aβ(1–40)-29.1 and Aβ(1–40)-51D) shows that the magnitude of the increase in toxicity associated with KW1 co-expression in each case correlated with the concentration of Aβ(1-40) (Figure 2D), as determined by western blot analysis of head homogenates (Additional file 1: Figure S4). Therefore, KW1 induces Aβ(1-40)-dependent neurotoxicity, although it does not discernibly affect other phenotypic properties, such as eye structure or climbing behaviour (Additional file 1: Figure S5A-C). The effect of KW1 is strikingly selective and co-expression with Aβ(1-42) and Aβ(1-42)-arc does not cause any significant change in lifespan compared to the expression of Aβ(1-42) or Aβ(1-42)-arc alone (Additional file 1: Figure S5D-I).

**Figure 3:**
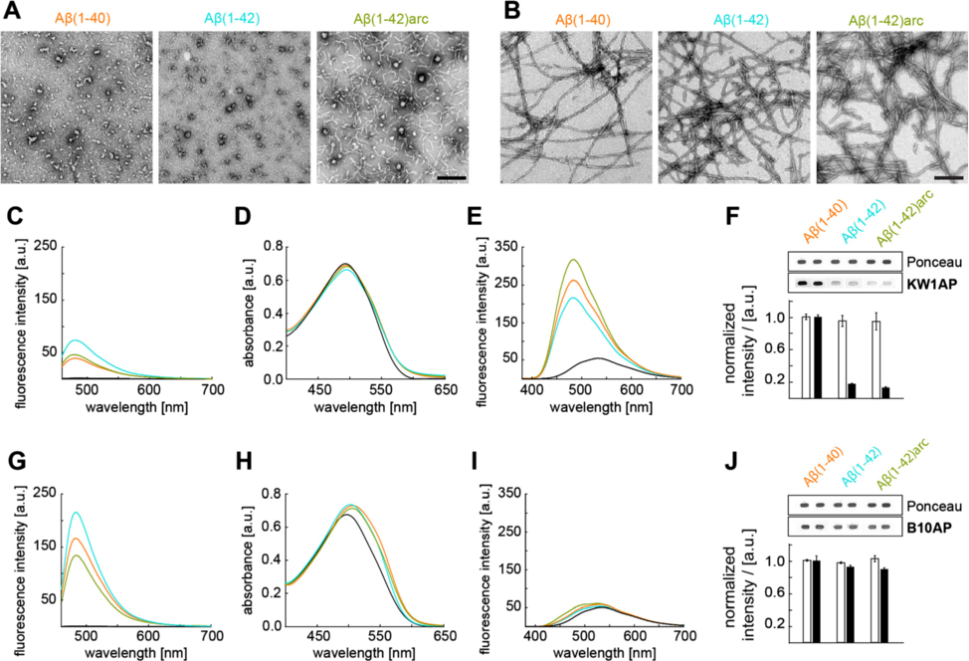
**B10 and KW1 binding to different Aβ structures. (A-B)** TEM images of intermediates **(A)** and amyloid fibrils **(B)** of the different Aβ variants as indicated. Scale bar represent 200 nm. Intermediates and fibrils are further characterized by interactions with Congo red **(C, G)**, Thioflavin T (ThT, **D, H**) and 8-anilinonaphthalene-1-sulfonate dyes (ANS, **E, I**). Measurements with fibrils were carried out in 50 mM sodium borate, pH 9.0. Reactions with intermediates contained 10% HFIP in water as the base solution. **(F)** Spot blot data of alkaline phosphatase (AP) coupled KW1 binding to intermediates. Densitometric quantifications and representative raw data stained with either Ponceau (loading control, white bars) or KW1AP (black bars). Aβ(1–40) Ponceau and KW1AP signals have been set to 100%. Error bars show standard deviation (n = 2–3). **(G-I)** Congo red, ThT and ANS binding of fibrils in 50 mM sodium borate buffer, pH 9.0. **(J)** Spot blot data of AP coupled B10 binding to fibrils. Densitometric quantifications and representative raw data shown. All panels show the same colour coding: black: solution without Aβ; ochre: Aβ(1–40); turquoise: Aβ(1–42); green: Aβ(1–42)arc.

While this selectivity fully agrees with *in vitro* data that KW1 preferentially interacts with Aβ(1-40) oligomers (Figure 3), it sharply contrast to results obtained upon co-expression of B10 together with Aβ in *Drosophila*, where we see no detectable phenotypic effects when compared to the expression of any of the Aβ peptides alone (Additional file 1: Figure S5). For all examined properties we find B10;Aβ40, B10;Aβ42 and B10;Aβ42arc flies to correspond to the respective lines Aβ40, Aβ42 and Aβ42arc. Although B10 is able to bind to all three fibril structures *in vitro* (Figure 3J) and although it perturbs fibril formation *in vitro*[30,35] and promotes protofibril formation at the expense of mature structures, fibril targeting with B10 does not affect the neurotoxicity of any of these peptides *in vivo*.

### KW1 selectively affects Aβ(1–40) peptide and alters its deposition in the brain

**Figure 4:**
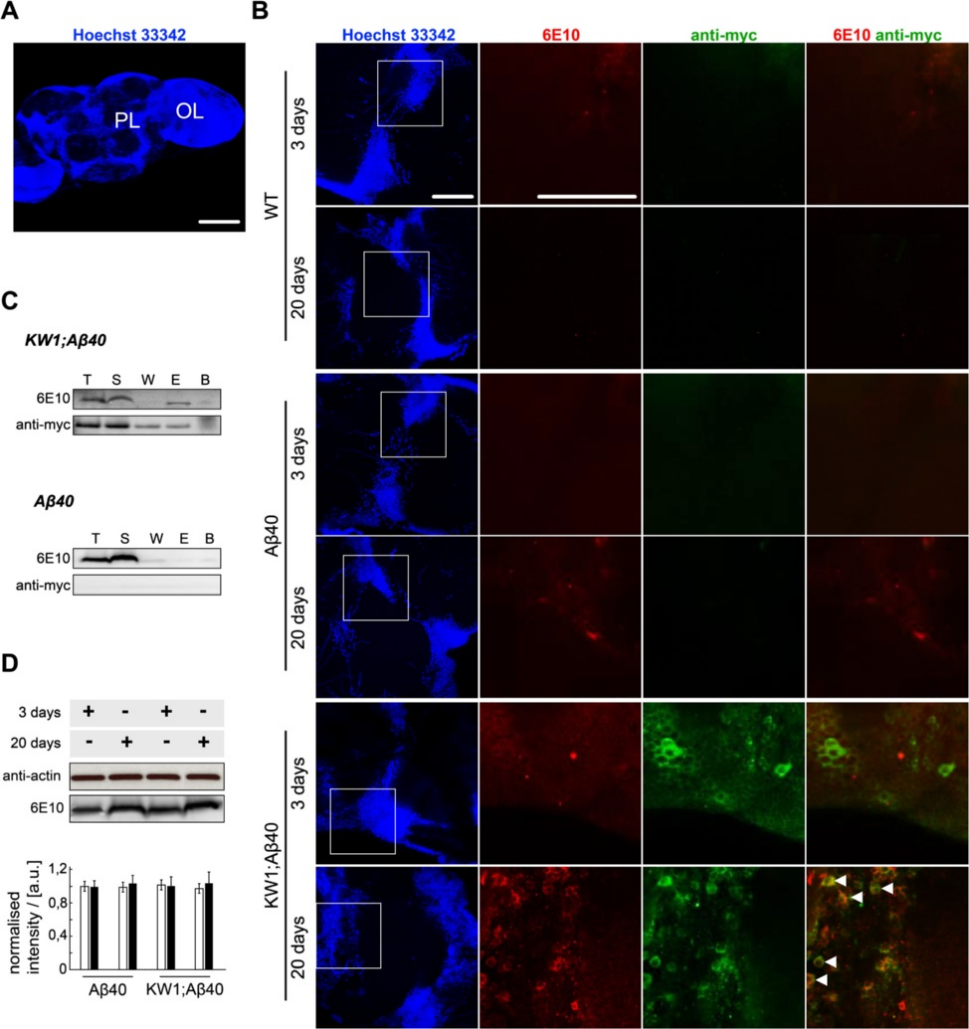
**KW1 interacts with Aβ(1–40) in fly tissue samples. (A)** Immunofluorescence microscopy image of the protocerebral lobe (PL) and optical lobe (OL) regions of WT *Drosophila* brain stained with Hoechst 33342 to visualize cell nuclei. **(B)** IFM images of adult brains from 3-day and 20-day old WT (top), Aβ40 (middle) and KW1;Aβ40 flies (bottom). Left column (blue): Hoechst 33342 staining. White boxes indicate the regions enlarged in the following three columns. Second column (red): Aβ-staining with 6E10 antibody. Third column (green): anti-myc antibody staining of myc-tagged B10 or KW1. Right column: overlay of the anti-myc and 6E10 signals. Arrows indicate signal co-localisation. Scale bars represent 50 μm **(A,B)**. **(C)** IP analysis of head homogenates from 20-day old KW1;Aβ40 and Aβ40 flies. Pull downs were performed against KW1. Resulting fractions were analyzed with WB to detect either Aβ (6E10) or myc-tagged KW1 (anti-myc). Abbreviations: T, total sample before IP; S, supernatant after incubation with beads; W, wash fraction; E, elution; B, beads after elution. **(D)** WB analysis with heads from 3-day and 20-day old Aβ40 and KW1;Aβ40 flies. Samples were probed with 6E10 to detect Aβ peptide (black bars). Anti-actin staining serves as loading control (white bars). All bars are normalised to the 3 days old Aβ40 sample (n = 2).

Aβ40 flies do not exhibit significant Aβ deposits by 20 days of age (Figure 4A,B), whereas co-expression of KW1 results in their appearance in age-matched flies (Figure 4B). KW1 partially co-localises with these foci and pull down of KW1 from KW1;Aβ40 head homogenates results in the co-immunoprecipitation (co-IP) of Aβ(1-40) (Figure 4C). While these data imply that KW1 affects the structure of Aβ concomitantly with inducing its toxicity, interactions must be rather transient in nature as we see no complete co-localisation between the two expression products. KW1 expression also does not significantly increase the steady state levels of Aβ(1-40) detected by western blotting of brain lysates of 3 or 20 days-old KW1;Aβ40 flies (Figure 4D), and KW1 primarily alters the spatial distribution and/or assembly state of Aβ(1–40) without dramatically changing its solubility.

Concerning B10, there was also no evidence that insufficient concentrations or a complete lack of interactions might explain the absence of effects of B10 expression on the flies. The ratio B10 to Aβ(1-42)-arc present in the fly brain (1:3) (Additional file 1: Figure S6) exceeds that at which B10 has been shown to be effective at suppressing Aβ fibril formation *in vitro* (as low as 1:10) [30]. Co-IP from fly head homogenates demonstrates that the two proteins are able to interact with one another (Additional file 1: Figure S4C), although there is only limited co-localization of the two expression products in B10;Aβ42arc flies (Additional file 1: Figure S4A, B).

### KW1 induces non-fibrillar aggregates with synaptotoxic activity

**Figure 5:**
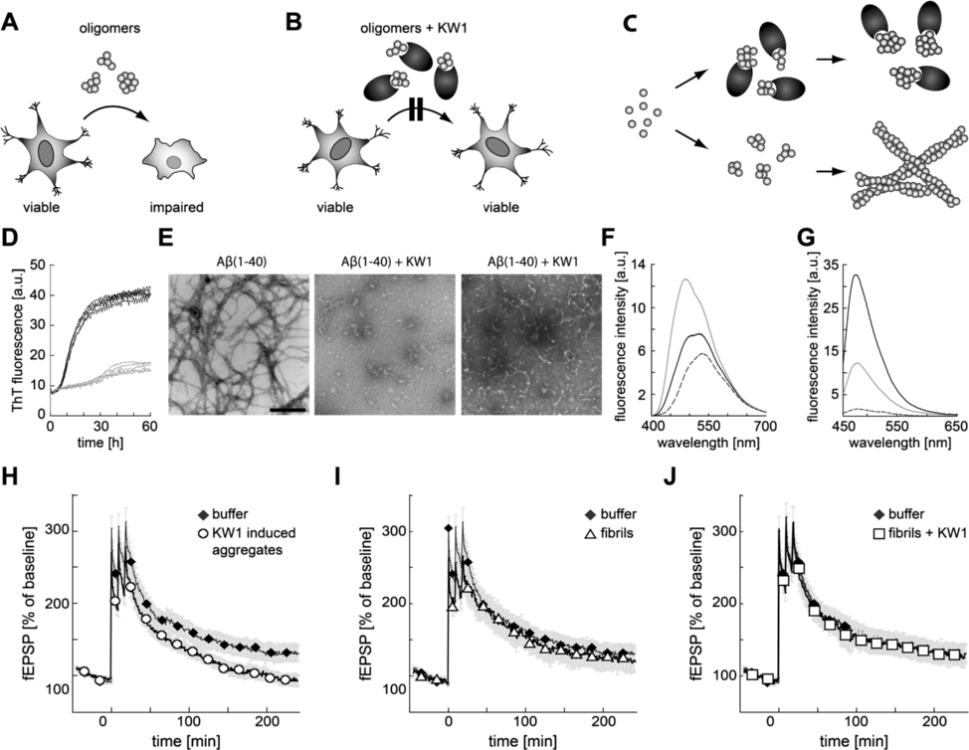
**Structural and biological effects of KW1 on Aβ(1–40) peptide *in vitro*. (A-C)** Hypothetical models of KW1 activity that are considered in this work; **(A-B)** KW1 antagonizes the toxic effect of oligomers. **(C)** KW1 modifies the Aβ assembly reaction. **(D)** Aggregation of Aβ(1–40) monitored by time-dependent ThT fluorescence at 490 nm. Black: Aβ(1–40) alone; grey: Aβ co-incubated with KW1 (n = 5). Length of the lag-phase: 5.6 ± 0.5 h (without KW1) and 24 ± 2.7 h (with KW1). **(E-G)** Structural comparison of Aβ fibrils, obtained by incubation of 100 μM Aβ(1–40) in 50 mM HEPES buffer, pH 7.4, 50 mM NaCl for 5 days at 37°C, and of KW1-stabilized aggregates, obtained by co-incubation of 100 μM Aβ(1–40) with 20 μM KW1 under the same conditions. **(E)** Negative stain TEM images. Different regions of the grid of KW1-induced aggregates are shown. Scale bar represents 200 nm. **(F)** ANS and **(G)** ThT fluorescence spectra recorded with KW1-stabilized Aβ aggregates (grey) or Aβ fibrils (black). Dashed spectra: dye without protein. **(H)** KW1-induced aggregates, obtained by co-incubation of Aβ(1–40) with KW1 for 5 days, potently reduces the LTP response (p = 0.029, estimated with repeated-measures ANOVA). **(I)** LTP measurement with slices pre-treated with Aβ(1–40) fibrils, obtained by a 5-day incubation of the pure peptide, shows no significant deviation from the buffer control. **(J)** Addition of KW1 to these fibrils 15 min prior to addition to the slice does not modify this result. Error bars (grey) represent standard error of the mean (n = 8-20).

The finding that KW1 expression specifically affects Aβ(1-40)-transgenic animals was highly surprising, given that it had previously been shown to antagonize Aβ(1-40) oligomer induced neurotoxicity (Figure 5A,B), as measured by the ability of preformed oligomers of Aβ(1-40) to disrupt synaptic plasticity and LTP in cultured murine brain slices [29,37,46]. An obvious contrast between the fly model described here, and previous *in vitro* work is that KW1 is produced in the brain of the fly concomitantly with Aβ(1-40) peptide and is present during fibril or oligomer formation (Figure 5C). Indeed, if we remodel such a situation *in vitro* by addition of KW1 to disaggregated Aβ(1-40) we find a significantly perturbed fibrillation pathway (Figure 5C). KW1 results in an extended lag phase and the formation of non-fibrillar species at the expense of mature fibrils (Figure 5D,E), similar to previous observations with dimeric KW1 [29]. Measured concentrations of KW1 (~1 μM) and Aβ(1–40) (~0.2 μM) in fly head homogenates (Additional file 1: Figure S4) translate into a 5:1 molar stoichiometry and are significantly larger than the sub-stoichiometric 1:5 molar ratio of KW1 to Aβ, which suffices to perturb Aβ(1–40) aggregation *in vitro* (Figure 5D,E).

The non-fibrillar aggregates formed by KW1 co-incubation with disaggregated Aβ(1–40) expose more surface hydrophobicity, as measured by increased binding of the dye 8-anilino-1-naphthalenesulfonic acid (ANS), than the more fibrillar aggregates formed in the absence of KW1 (Figure 5F). They also bind Thioflavin T (ThT), a dye that interacts with ordered β-sheet rich aggregates, more weakly than the more fibrillar species formed in the absence of KW1 (Figure 5G).

As surface exposed hydrophobics and weak ThT binding were previously described as a signature of toxic amyloid aggregates [47,48], we sought to determine by LTP as to whether or not the neurotoxic effects of expressing KW1 with Aβ(1-40) *in vivo* can be rationalised by the ability of KW1 to promote the formation of hydrophobic non-fibrillar Aβ(1–40) aggregates. We applied different Aβ(1-40) preparations to the slice for 2 hours prior to electrophysiology recordings. We found that a 2 hour pre-incubation of the slices with 5-day old non-fibrillar Aβ(1–40) aggregates formed in the presence of KW1, prior to their addition to the culture, significantly inhibits LTP compared to untreated cultures (Figure 5H). The synaptic potentiation measured at 225 min was 102% ± 9 field excitatory postsynaptic potential (fEPSP) with KW1-induced Aβ(1–40) aggregates and 141% ± 10 fEPSP in the buffer treated control. No significant effect on the establishment of LTP is observed, if slices are pre-incubated for 2 hours with 5-day old Aβ(1–40) fibrils formed in the absence of KW1 (Figure 5I) or if KW1 was added to the fibrils after their formation but prior to their addition to the slice cultures (Figure 5J).

**Figure 6:**
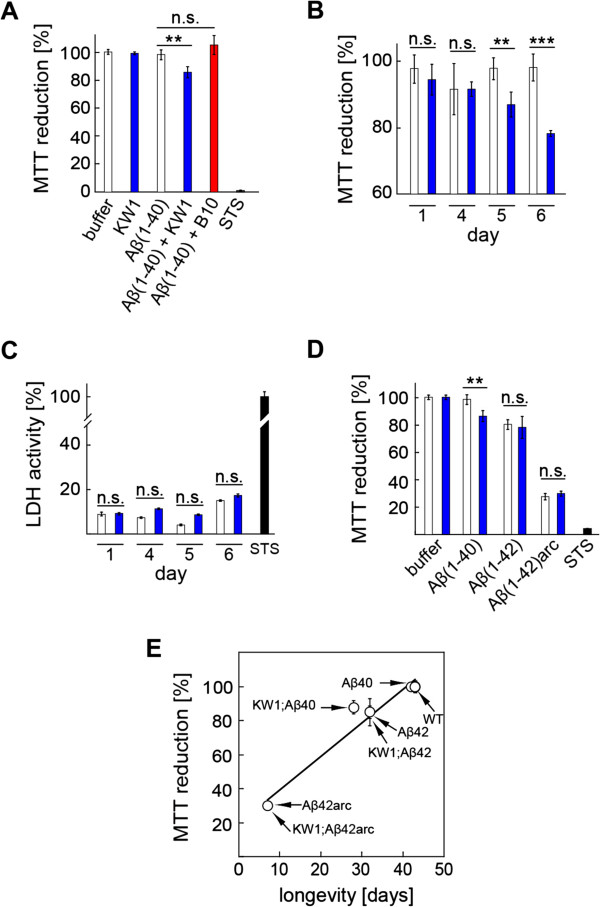
**Effect of KW1 on the metabolic activity of Aβ-species on SH-SY5Y cells. (A)** MTT assay of SH-SY5Y cells treated with Aβ(1–40) aggregates after 5 days of incubation with or without antibody fragments at 5:1 molar ratio of Aβ versus B10 or KW1. Molar ratio refers to the polypeptide chains. Cells treated with buffer were set to 100%. The general cell toxin staurosporine (STS, 2 μM) served as a positive control. **(B,C)** MTT-reduction **(B)** and LDH assay **(C)** of SH-SY5Y cells treated with Aβ(1–40) incubated for different periods of time with KW1 (blue) or without (white). SSP-treated cells defined 100% LDH activity. **(D)** MTT assay of SH-SY5Y cells treated with Aβ(1–40), Aβ(1–42) or Aβ(1–42)arc peptides incubated in 50 mM HEPES buffer, pH 7.4, 50 mM NaCl at 37°C for 5 days with KW1 (blue) or without (white). n.s.: non-significant, **: p < 0.01, ***: p < 0.001. Error bars represent standard deviation of n = 6. **(E)** Corresponding Aβ isoforms with or without KW1 plotted based on original life span data (Table 1) and MTT reduction *in vitro* (Figure 6). Please note the overlap of several data points. The strong correlation between MTT reduction and neurotoxicity *in vivo* fits with linear regression (R = 0.98).

We confirmed these effects for a human neuronal system by adding these same Aβ(1-40)/KW1 preparations to human neuroblastoma (SH-SY5Y) cells and measurement of their effects on neuronal metabolic activity with the 3-(4,5-dimethylthiazol-2-yl)-2,5-diphenyltetrazolium bromide (MTT) assay. Co-incubation of Aβ(1-40) with KW1 throughout the aggregation reaction generated aggregates that modulate the activity SH-SY5Y cells and induce a marked ~12% reduction of the MTT value (Figure 6A), whereas those formed in the absence of KW1 or in the presence of B10 did not (Figure 6A). Notably, the activity of Aβ(1-40) when co-incubated with KW1 increased with the length of the incubation time prior to their application to the cultured cells (from 1 to 5 days). This observation is in full accord with the effect of KW1 being to promote the progressive accumulation of neurotoxic forms of Aβ(1-40) aggregates over this time scale (Figure 6B). These effects of combinations of KW1 and Aβ(1–40) on neuronal metabolic activity were significantly smaller than those observed following treatment with Aβ(1–42) and Aβ(1–42)arc peptides (Figure 6D). In line with other experimental paradigms reported here, the activities of the latter were also not modifiable by KW1 and did not depend on the presence or absence of this antibody fragment during aggregation.

While MTT does not directly monitor neuronal death in these experiments, lactate dehydrogenase (LDH) assay, which measures membrane disruption and thus reports more directly on cellular toxicity than MTT, shows no significant effects of Aβ on SH-SY5Y cells (Figure 6C), we find a striking correlation of the MTT-effects and fly toxicity *in vivo* (Figure 6E). Therefore, pure peptides are able to form species *in vitro* that capture activities associated with Aβ pathogenicity *in vivo*. They further testify to the existence of different populations of Aβ assemblies inside the fly that differ in the chemical structure of Aβ peptide, formation mechanism and sensitivity towards modulation by KW1; that is, there is no uniquely active Aβ structure but a collection of toxic Aβ structures *in vitro* and *in vivo*.

## Discussion

In this study we show that conformational targeting is a suitable approach to probe the process of Aβ aggregation and its neurotoxic consequences in an intact living organism, the fruit fly. Previous research already established that targeting of Aβ monomers *in vivo* with an artificial Affibody binding protein (analogous to the antibody fragments described here) is able to interfere with Aβ toxicity *in vivo*[49] and so we sought to extend this experimental design in the present study to investigate multimeric peptide assemblies of Aβ in a similar manner. Our results reveal a striking contrast between the effects of targeting oligomeric and fibrillar assemblies of the Aβ peptide *in vivo*. Whilst the fibril-binding B10 antibody fragment is unable to perturb the neurotoxicity associated with any of the forms of Aβ studied here, the oligomer-specific KW1 antibody fragment shows a potent and highly specific effect and alters toxicity in the fly model. This observation is consistent with the view that oligomeric intermediates are more toxic than mature fibrils.

Unexpectedly, KW1 promotes rather than neutralizes toxicity. This finding is in sharp contrast to many previous studies, which report oligomer-binding conformational ligands to antagonize the detrimental effects of oligomers (Figure 5A,B), presumably by preventing their binding to cellular surfaces or receptors. These previous studies usually used an experimental set up where the disturbance of ordered neuronal functions was recorded in response to preformed toxic oligomers. Thus, addition of an oligomer-specific binder to this system interfered with this process and blocked the effects of oligomers on neurons [50-56]. If KW1 is explored under such a set-up, for example by using LTP measurements as the readout of toxicity, we find oligomeric activity to be blocked [29], similar to many other oligomer-specific ligands.

This test system, however, does not capture the possibility that an oligomer-binder can affect the peptide during aggregates, which is physiologically relevant as KW1 is present in the fly together with Aβ(1–40) peptide for prolonged periods of time. If we remodel this situation *in vitro* by allowing Aβ(1–40) peptide to aggregate in the presence of KW1, we obtain a result that is now fully consistent with the fly data and demonstrates an increased Aβ activity in both LTP (Figure 5H) and MTT assays (Figure 6A). The mechanism underlying these effects appears therefore to involve interference by the oligomer-specific binder KW1 with the process of the assembly of Aβ peptides into amyloid structures (Figure 5C). This effect could result from a variety of processes, such as the suppression of the late stages of the aggregation reaction through binding to early intermediates or interaction with late-stage intermediates such that non-fibrillar Aβ assemblies prevail (Figure 5E). Indeed, the effects of KW1 are also associated with an altered structure of the Aβ peptide that is evident from TEM (Figure 5E) and ANS binding experiments (Figure 5F). Moreover, it appears that the Aβ(1–40) peptide only becomes toxic under certain conditions, such as is observed here in the presence of KW1 as a cofactor.

Another highly remarkable finding from our fly studies is that KW1 selectively affected Aβ(1–40)-expressing flies and mirrors the *in vitro* observation that KW1 preferentially interacts with Aβ(1–40)-derived oligomers compared with Aβ(1–42) or Aβ(1–42)-arc-derived intermediates (Additional file 1: Figure S5A, D, G). That is, there must be at least subtle differences in the structure or surface texture of these states and also, as Aβ toxicity is not affected by KW1 in all fly models, in the mechanisms by which they form.

These data have significance for the design of Aβ-modifying therapeutic strategies for the treatment of Alzheimer’s disease. First, if Aβ-dependent AD pathology does not only depend on a single Aβ state, strategies to bind and to neutralize Aβ peptide more generally or to simultaneously prevent formation of multiple peptide assembly states may be preferable for achieving efficient therapeutic design rather than targeting a single, though highly toxic, molecular state. Second, the biological activity of state-specific binders might be highly context-dependent, as may not only block the binding of oligomers to their cellular receptors, but also modulate the peptide self-assembly reaction. Therefore, the biological consequences of a binder are difficult to predict *a priori*, and the same protein, KW1, can either prevent or enhance the neurotoxicity of Aβ(1-40) depending on the time point in the aggregation process at which it is added. We believe that this effect could be in particular a problem of approaches that solely aim to target oligomers in order to antagonize their activity by binding. In the case of proteins with additional activities, such as full-blown antibodies with functional Fc-parts, however, these effects could be overruled as they may alert the immune system to induce the specific destruction of the bound ligand. This explains why oligomer-specific antibodies may be functional *in vivo*[53,55].

## Conclusions

Understanding the kinetics and underlying mechanisms of Aβ aggregation in the brains or patients suffering from AD, and the balance of these process with those that facilitate degradation and clearance of aggregates, is crucial for maximising the efficacy of therapeutic strategies based on the modulation of Aβ aggregation. Conformational targeting with appropriately designed antibody fragments is not only a way to determine the types of aggregates that are present in the intact nervous system of a living animal, but also to analyse the functional effects arising from specific perturbations of the mechanisms of Aβ peptide aggregation *in vivo*. By combining these studies with *in vitro* analysis of the biophysical properties of the antibody fragments and their interactions with Aβ, it is possible to provide significant insights into the molecular basis of the pathogenesis of conformational diseases and perhaps to generate successful strategies, or to detect potential pitfalls, when devising therapeutic solutions to these devastating diseases.

## Competing interests

All authors declare that there are no competing interests.

## Authors’ contributions

JW, RR, MW, MW and LML carried out research, KGR, CMD, UH, DCC, LML and MF designed research, JW, MW, LML. and MF wrote the paper. All authors read and approved the final manuscript.

## Supplementary Material

Additional file 1: Figure S1 Amino acid sequences of the expressed polypeptide chains. **Figure S2.** B10AP and KW1AP do not cross-react with *Drosophila* proteins. **Figure S3. ***Drosophila* Schneider S2 cells are able to express functional KW1 and B10 antibody fragments. **Figure S4.** Phenotypic effects of KW1 and B10 expression on Aβ-transgenic flies. **Figure S5.** B10 interacts with Aβ peptide in tissue samples. **Figure S6.** Concentrations of Aβ, B10 and KW1 in fly head homogenates. Click here for file

## References

[B1] Eisenberg D Jucker M The amyloid state of proteins in human diseases Cell 2012 2 1188 1203 10.1016/j.cell.2012.02.022 22424229 PMC3353745

[B2] Bellotti V Mangione P Merlini G Molecular mechanisms of amyloidosis N Engl J Med 2003 2 583 596 10.1056/NEJMra023144 12904524

[B3] Chiti F Dobson CM Protein misfolding, functional amyloid, and human disease Annu Rev Biochem 2006 2 333 366 10.1146/annurev.biochem.75.101304.123901 16756495

[B4] Eichner T Radford SE A diversity of assembly mechanisms of a generic amyloid fold Mol Cell 2011 2 8 18 10.1016/j.molcel.2011.05.012 21726806

[B5] Dodel RC Hampel H Du YS Immunotherapy for Alzheimer’s disease Lancet Neurol 2003 2 215 220 10.1016/S1474-4422(03)00349-1 12849209

[B6] Haass C Selkoe DJ Soluble protein oligomers in neurodegeneration: lessons from the Alzheimer’s amyloid bold beta-peptide Nat Rev Mol Cell Biol 2007 2 101 112 10.1038/nrm2101 17245412

[B7] Ittner LM Götz J Amyloid-β and tau–a toxic pas de deux in Alzheimer’s disease Nat Rev Neurosci 2011 2 65 72 21193853 10.1038/nrn2967

[B8] Walsh DM Teplow DB Alzheimer’s disease and the amyloid β protein Prog Mol Biol Tranls 2012 2 101 124 10.1016/B978-0-12-385883-2.00012-6 22482449

[B9] Morgado I Fändrich M Review: assembly of Alzheimer’s Aβ peptide into nanostructured amyloid fibrils Curr Opin Colloid In 2011 2 508 514 10.1016/j.cocis.2011.06.016

[B10] Nilsberth C Westlind-Danielsson A Eckman CB Condron MM Axelman K Forsell C Stenh C Luthman J Teplow DB Younkin SG Näslund J Lannfelt L The ‘Arctic’ APP mutation (E693G) causes Alzheimer’s disease by enhanced Abeta protofibril formation Nat Neurosci 2001 2 887 893 10.1038/nn0901-887 11528419

[B11] Schilling S Zeitschel U Hoffmann T Heiser U Francke M Kehlen A Holzer M Birgit Hutter-Paier B Prokesch M Windisch M Jagla W Schlenzig D Lindner C Rudolph T Reuter G Cynis H Montag D Demuth HU Rossner S Glutaminyl cyclase inhibition attenuates pyroglutamate Abold beta and Alzheimer’s disease–like pathology Nat Med 2008 2 1106 1111 10.1038/nm.1872 18836460

[B12] Lashuel HA Lansbury PT Jr Are amyloid diseases caused by protein aggregates that mimic bacterial pore-forming toxins? Q Rev Biophys 2006 2 167 201 10.1017/S0033583506004422 16978447

[B13] Cherny I Gazit E Amyloids: not only pathological agents but also ordered nanomaterials Angew Chem Int Edit 2008 2 4062 4069 10.1002/anie.200703133 18412209

[B14] Fändrich M Oligomeric intermediates in amyloid formation: structure determination and mechanisms of toxicity J Mol Biol 2012 2 427 440 10.1016/j.jmb.2012.01.006 22248587

[B15] Lesne S Koh MT Kotilinek L Kayed R Glabe CG Yang A Gallagher M Ashe KH A specific amyloid-β protein assembly in the brain impairs memory Nature 2005 2 352 357 10.1038/nature04533 16541076

[B16] Martins IC Kuperstein I Wilkinson H Maes E Vanbrabant M Lipids revert inert Abeta amyloid fibrils to neurotoxic protofibrils that affect learning in mice EMBO J 2008 2 224 233 10.1038/sj.emboj.7601953 18059472 PMC2206134

[B17] Glabe CG Conformation-dependent antibodies target diseases of protein misfolding Trends Biochem Sci 2004 2 542 547 10.1016/j.tibs.2004.08.009 15450609

[B18] O’Nuallain B Ronald Wetzel R Conformational Abs recognizing a generic amyloid fibril epitope Proc Natl Acad Sci U S A 2002 2 1485 1490 10.1073/pnas.022662599 11818542 PMC122217

[B19] Perchiacca JM Ladiwala AR Bhattacharya M Tessier PM Structure-based design of conformation- and sequence-specific antibodies against amyloid β Proc Natl Acad Sci U S A 2012 2 84 89 10.1073/pnas.1111232108 22171009 PMC3252897

[B20] Nerelius C Sandegren A Sargsyan H Raunak R Leijonmarck H Chatterjee U Fisahn A Imarisio S Lomas DA Crowther DC Strömberg R Johansson J Alpha-helix targeting reduces amyloid-beta peptide toxicity Proc Natl Acad Sci U S A 2009 2 9191 9196 10.1073/pnas.0810364106 19458258 PMC2695042

[B21] Kvam E Nannenga BL Wang MS Jia Z Sierks MR Messer A Conformational targeting of fibrillar polyglutamine proteins in live cells escalates aggregation and cytotoxicity PLoS ONE 2009 2 5 e5727 doi:10.1371/journal.pone.0005727 10.1371/journal.pone.0005727 19492089 PMC2683928

[B22] Crowther DC Kinghorn KJ Miranda E Page R Curry JA Duthie FA Gubb DC Lomas DA Intraneuronal Abeta, non-amyloid aggregates and neurodegeneration in a Drosophila model of Alzheimer’s disease Neuroscience 2005 2 123 135 10.1016/j.neuroscience.2004.12.025 15780472

[B23] Luheshi LM Tartaglia GG Brorsson AC Pawar AP Watson IE Chiti F Vendruscolo M Lomas DA Dobson CM Crowther DC Systematic in vivo analysis of the intrinsic determinants of amyloid Beta pathogenicity PLoS Biol 2007 2 11 e290 doi:10.1371/journal.pbio.0050290 10.1371/journal.pbio.0050290 17973577 PMC2043051

[B24] Rival T Page RM Chandraratna DS Sendall TJ Ryder E Liu B Lewis H Rosahl T Hider R Camargo LM Shearman MS Crowther DC Lomas DA Fenton chemistry and oxidative stress mediate the toxicity of the beta-amyloid peptide in a Drosophila model of Alzheimer’s disease Eur J Neurosci 2009 2 1335 1347 10.1111/j.1460-9568.2009.06701.x 19519625 PMC2777252

[B25] Liu B Moloney A Meehan S Morris K Thomas SE Serpell LC Hider R Marciniak SJ Lomas DA Crowther DC Iron promotes the toxicity of amyloid beta peptide by impeding its ordered aggregation J Biol Chem 2011 2 4248 4256 10.1074/jbc.M110.158980 21147772 PMC3039358

[B26] Sofola O Kerr F RogersI KK Augustin H Gandy C Allen MJ Hardy J Lovestone S Partridge L Inhibition of GSK-3 ameliorates Aβ pathology in an adult-onset Drosophila model of Alzheimer’s disease PLoS Genet 2010 2 9 e1001087 doi:10.1371/journal.pgen.1001087 10.1371/journal.pgen.1001087 20824130 PMC2932684

[B27] Auluck PK Caraveo G Lindquist S α-Synuclein: membrane interactions and toxicity in Parkinson’s disease Annu Rev Cell Dev Biol 2010 2 211 233 10.1146/annurev.cellbio.042308.113313 20500090

[B28] Wittmann CW Wszolek MF Shulman JM Salvaterra PM Lewis J Hutton M Feany MB Tauopathy in Drosophila: neurodegeneration without neurofibrillary tangles Science 2001 2 711 714 10.1126/science.1062382 11408621

[B29] Morgado I Wieligmann K Bereza M Rönicke R Meinhardt K Annamalai K Baumann M Wacker J Hortschansky P Malešević M Parthier C Mawrin C Schiene-Fischer C Reymann KG Stubbs MT Balbach J Görlach M Horn U Fändrich M Molecular basis of β-amyloid oligomer recognition with a conformational antibody fragment Proc Natl Acad Sci U S A 2012 2 12503 12508 10.1073/pnas.1206433109 22814377 PMC3412029

[B30] Habicht G Haupt C Friedrich RP Hortschansky P Sachse C Meinhardt J Wieligmann K Gellermann GP Brodhun M Götz J Halbhuber KJ Röcken C Horn U Fändrich M Directed selection of a conformational antibody domain that prevents mature amyloid fibril formation by stabilizing Aβ protofibrils Proc Natl Acad Sci U S A 2007 2 19232 19237 10.1073/pnas.0703793104 18042730 PMC2148273

[B31] Haupt C Morgado I Kumar ST Parthier C Bereza M Hortschansky P Stubbs MT Horn U Fändrich M Amyloid fibril recognition with the conformational B10 antibody fragment depends on electrostatic interactions J Mol Biol 2011 2 341 348 10.1016/j.jmb.2010.10.059 21059358

[B32] Haupt C Bereza M Kumar ST Kieninger B Morgado I Hortschansky P Fritz G Röcken C Horn U Fändrich M Pattern recognition with a fibril-specific antibody fragment reveals the surface variability of natural amyloid fibrils J Mol Biol 2011 2 529 540 10.1016/j.jmb.2011.02.032 21376731

[B33] Upadhaya AR Lungrin I Yamaguchi H Fändrich M Thal DR High-molecular weight Aβ oligomers and protofibrils are the predominant Aβ species in the native soluble protein fraction of the AD brain J Cell Mol Med 2012 2 287 295 10.1111/j.1582-4934.2011.01306.x 21418518 PMC3823292

[B34] Kieninger B Gioeva Z Krüger S Westermark GT Friedrich RP Fändrich M Röcken C PTAA and B10: new approaches to amyloid detection in tissue-evaluation of amyloid detection in tissue with a conjugated polyelectrolyte and a fibril-specific antibody fragment Amyloid 2011 2 47 52 10.3109/13506129.2011.560623 21401323

[B35] Scheidt HA Morgado I Rothemund S Huster D Fändrich M Solid-state NMR spectroscopic investigation of Aβ protofibrils: implication of a β-sheet remodeling upon maturation into terminal amyloid fibrils Angew Chem Int Ed Engl 2011 2 2837 2840 10.1002/anie.201007265 21387500

[B36] Hortschansky P Schroeckh V Christopeit T Zandomeneghi G Fändrich M The aggregation kinetics of Alzheimer’s β-amyloid peptide is controlled by stochastic nucleation Prot Sci 2005 2 1753 1759 10.1110/ps.041266605 PMC2253354 15937275

[B37] Haupt C Leppert J Rönicke R Meinhardt J Yadav JK Ramachandran R Ohlenschläger O Reymann KG Görlach M Fändrich M Structural basis of β-amyloid-dependent synaptic dysfunctions Angew Chem Int Ed 2012 2 1576 1577 10.1002/anie.201105638 22234970

[B38] Bischof K An optimized transgenesis system for Drosophila using germ-line-specific φC31 integrases Proc Natl Acad Sci U S A 2007 2 3312 3317 10.1073/pnas.0611511104 17360644 PMC1805588

[B39] Green C Levashina E McKimmie C Dafforn T Reichhart JM Gubb D The necrotic gene in Drosophila corresponds to one of a cluster of three serpin transcripts mapping at 43A1.2 Genetics 2000 2 1117 1127 11063688 10.1093/genetics/156.3.1117 PMC1461326

[B40] Brand AH Perrimon N Targeted gene expression as a means of altering cell fates and generating dominant phenotypes Development 1993 2 401 415 8223268 10.1242/dev.118.2.401

[B41] Speretta E Jahn TR Tartaglia GG Favrin G Barros TP Imarisio S Lomas DA Luheshi LM Crowther DC Dobson CM Expression in drosophila of tandem amyloid beta peptides provides insights into links between aggregation and neurotoxicity J Biol Chem 2012 2 20748 20754 10.1074/jbc.M112.350124 22461632 PMC3370257

[B42] Ashburner M Golic KG Hawley RS Drosophila: a laboratory handbook 2005 2 Cold Spring Harbor, New York: Cold Spring Harbor Laborators Press XXVIII 1408pp

[B43] Christopeit T Hortschansky P Schroeckh V Gührs K Zandomeneghi G Fändrich M Mutagenic analysis of the nucleation propensity of oxidized Alzheimer’s β-amyloid peptide Protein Sci 2005 2 2125 2131 10.1110/ps.051470405 15987892 PMC2279324

[B44] Gill SC von Hippel PH Calculation of protein extinction coefficients from amino acid sequence data Anal Biochem 1989 2 319 326 10.1016/0003-2697(89)90602-7 2610349

[B45] Rönicke R Mikhaylova M Rönicke S Meinhardt J Schröder UH Fändrich M Reiser G Kreutz MR Reymann KG Early neuronal dysfunction by amyloid β oligomers depends on activation of NR2B-containing NMDA receptors Neurobiol Aging 2011 2 2219 2228 10.1016/j.neurobiolaging.2010.01.011 20133015

[B46] Rönicke R Klemm A Meinhardt J Schröder UH Fändrich M Reymann KG Abeta mediated diminution of MTT reduction–an artefact of single cell culture? PLoS ONE 2008 2 9 e3236 doi:10.1371/journal.pone.0003236 10.1371/journal.pone.0003236 18800168 PMC2529401

[B47] Bolognesi B Kumita JR Barros TP Esbjorner EK Luheshi LM Crowther DC Wilson MR Dobson CM Favrin G Yerbury JJ ANS binding reveals common features of cytotoxic amyloid species ACS Chem Biol 2010 2 735 740 10.1021/cb1001203 20550130

[B48] Krishnan R Goodman JL Mukhopadhyay S Pacheco CD Lemke EA Deniz AA Lindquist S Conserved features of intermediates in amyloid assembly determine their benign or toxic states Proc Natl Acad Sci U S A 2012 2 11172 11177 10.1073/pnas.1209527109 22745165 PMC3396487

[B49] Luheshi LM Hoyer W Pereira de Barros T van Dijk HI Brorsson AC Macao B Persson C Crowther DC Lomas DA Stahl S Dobson CM Härd T Sequestration of the Abeta peptide prevents toxicity and promotes degradation in vivo PLoS Biol 2010 2 3 e1000334 doi:10.1371/journal.pbio.1000334 10.1371/journal.pbio.1000334 20305716 PMC2838747

[B50] Wang XP Zhang JH Wang YJ Feng Y Zhang X Sun XX Li JL Du XT Lambert MP Yang SG Zhao M Klein WL Liu RT Conformation-dependent single-chain variable fragment antibodies specifically recognize beta-amyloid oligomers FEBS Lett 2009 2 579 584 10.1016/j.febslet.2008.12.064 19162022

[B51] Lambert MP Viola KL Chromy BA Chang L Morgan TE Yu J Venton DL Krafft GA Finch CE Klein WL Vaccination with soluble Aβ oligomers generates toxicity-neutralizing antibodies J Neurochem 2001 2 595 605 11701763 10.1046/j.1471-4159.2001.00592.x

[B52] Kayed R Head E Thompson JL McIntire TM Milton SC Cotman CW Glabe CG Common structure of soluble amyloid oligomers implies common mechanism of pathogenesis Science 2003 2 486 489 10.1126/science.1079469 12702875

[B53] Lee EB Leng LZ Zhang B Kwong L Trojanowski JQ Abel T Lee VM Targeting amyloid-beta peptide (Abeta) oligomers by passive immunization with a conformation-selective monoclonal antibody improves learning and memory in Abeta precursor protein (APP) transgenic mice J Biol Chem 2006 2 4292 4299 10.1074/jbc.M511018200 16361260

[B54] Goñi F Prelli F Ji Y Scholtzova H Yang J Sun Y Liang FX Kascsak R Kascsak R Mehta P Wisniewski T Immunomodulation targeting abnormal protein conformation reduces pathology in a mouse model of Alzheimer’s disease PLoS ONE 2010 2 10 e13391 doi:10.1371/journal.pone.0013391 10.1371/journal.pone.0013391 20967130 PMC2954195

[B55] Hillen H Barghorn S Striebinger A Labkovsky B Müller R Nimmrich V Nolte MW Perez-Cruz C van der Auwera I van Leuven F van Gaalen M Bespalov AY Schoemaker H Sullivan JP Ebert U Generation and therapeutic efficacy of highly oligomer-specific beta-amyloid antibodies J Neurosci 2010 2 10369 10379 10.1523/JNEUROSCI.5721-09.2010 20685980 PMC6634649

[B56] Shughrue PJ Acton PJ Breese RS Zhao WQ Chen-Dodson E Hepler RW Wolfe AL Matthews M Heidecker GJ Joyce JG Villarreal SA Kinney GG Anti-ADDL antibodies differentially block oligomer binding to hippocampal neurons Neurobiol Aging 2010 2 189 202 10.1016/j.neurobiolaging.2008.04.003 18486276

