# Supplementary material for: Oligomer-targeting with a conformational antibody fragment promotes toxicity in Aβ-expressing flies
Source: Acta Neuropathol Commun. 2014 Apr 11;2:43. doi: 10.1186/2051-5960-2-43 (PMC4029271; doi:10.1186/2051-5960-2-43)
Supplement: Additional file 1: Figure S1 — Amino acid sequences of the expressed polypeptide chains. Figure S2. B10AP and KW1AP do not cross-react with Drosophila proteins. Figure S3. Drosophila Schneider S2 cells are able to express functional KW1 and B10 antibody fragments. Figure S4. Phenotypic effects of KW1 and B10 expression on Aβ-transgenic flies. Figure S5. B10 interacts with Aβ peptide in tissue samples. Figure S6. Concentrations of Aβ, B10 and KW1 in fly head homogenates. [file 2051-5960-2-43-S1.PDF]

Supporting Information

Oligomer-Targeting with a Conformational Antibody Fragment Promotes Toxicity in Aβ-expressing flies

Jessica Wacker[1,#], Raik Rönicke[2], Martin Westermann[3], Melanie Wulff[1,4], Klaus G. Reymann[2], Christopher M. Dobson[5], Uwe Horn[6], Damian C. Crowther[7,8], Leila M. Luheshi[5], Marcus Fändrich[1,4]

1: Max-Planck Research Unit for Enzymology of Protein Folding, 06120 Halle (Saale), Germany

2: German Centre for Neurodegenerative Diseases (DZNE), 39118 Magdeburg, Germany

3: Electron Microscopy Center, Jena University Hospital, Friedrich Schiller University Jena, 07743 Jena, Germany

4: Ulm University, Institute for Pharmaceutical Biotechnology, Helmholtzstr. 8/1, 89081 Ulm, Germany

5: Department of Chemistry, University of Cambridge, Cambridge CB2 1EW, U. K.

6: Leibniz Institut für Naturstoff-Forschung und Infektionsbiologie, Hans-Knöll-Institut, 07745 Jena, Germany

7: Department of Genetics, University of Cambridge, Cambridge CB2 3EH, U. K.

8: Cambridge Institute for Medical Research, Cambridge, CB2 0XY, U.K.

Supporting Methods

Western blot (WB)

Unless indicated otherwise, gels were blotted onto Protran BA 79 cellulose nitrate membranes (0.1 µm; Schleicher and Schuell). Membranes were blocked for 1 h in 5 % low fat milk powder in PBS and incubated with the primary antibody in PBST (PBS + 0.05 % TritonX 100) by overnight incubation at 4°C. The following primary antibodies were used: 6E10 (monoclonal, mouse, recognizes residues 1-16 of Aβ peptide, Covance, 1:1000) ab8224 (monoclonal, mouse, recognized β-actin, Abcam, 1:1000) or ab9106 (polyclonal, rabbit, myc-tag specific, Abcam, 1:1000). Unbound primary antibodies were removed by washing the membrane three times for 5 min in PBST. Bound primary antibodies were detected with goat anti-mouse or goat anti-rabbit secondary antibodies, which were conjugated with horseradish peroxidase (Dako, Denmark, 1:1000 in PBST). Horseradish peroxidase was detected using the SuperSignal West Chemiluminescent Substrate (Pierce). Aβ WB involved a 5 min boiling step of the nitrocellulose membranes in PBS after electro blotting to increase the efficiency of detection.

Purification of KW1AP and B10AP from **Drosophila** *Schneider S2 cells*

For expression of B10AP and KW1AP, Schneider S2 cells were seeded at a density of 10^6 cells/ml into a 6-well plate containing 3 ml Insect Express Sf9-S2 medium (PAA Laboratories) per well. The cells were grown for 5 hours at 28°C, before we added 100 µl of the transfection mix. This solution was prepared by mixing 0,4 µg pCoBlast (selection vector) with 15 µl FuGENE® HD Transfection Reagent (Roche), 4 µg transgenic vector (pMT/V5-HisA-B10AP-S2 or pMT/V5-HisA-KW1-S2) and sterile water. In these vectors KW1 and B10 were directly expressed as AP fusion proteins to aid in detection (SI Fig. 1B). Addition of a his-tag affords the facile enrichment of the resulting proteins, termed B10AP-S2 and KW1AP-S2, via nickel

chelate chromatography (SI Fig. 3). The solution was briefly spun down to collect the solution in the tube, before it was incubated for 15 min at room temperature. This solution was added drop-wise and under gentle agitation to the S2 cells. After a 48-h incubation period (28°C), we added 30 µg/ml blasticidine to the culture supernatant in order to select for stably transfected cells.

To induce protein expression we seeded the cells at a density of 10^6 cells/ml and allowed them to grow at 28°C until they had reached the onset of the log-phase. We then added Cu_2SO_4 to a final concentration of 1 mM, and after further incubation for 30 more hours, the cells were spun down at 13.000 × *g* (10 min, 4°C) and the proteins were purified from the supernatant. To that end, we first removed all copper ions by dialysis against a 10-fold volume of buffer A (50 mM sodium phosphate pH 8.0; 300 mM NaCl; 50 mM imidazol). We used a total of 4 dialysis steps (Spectra/Por® 4 membrane, molecular weight cut off 12-14 kDa, Roth), each lasting for 4 h (4°C). The remaining solution was then *applied onto a Nickel Sepharose Fast Flow (GE Healthcare) column operated by an ÄKTA Explorer system (GE Healthcare), and the protein was eluted with a step gradient from 5 % to 100 % buffer B (*50 mM sodium phosphate pH 8.0; 300 mM NaCl; 250 mM imidazol). The protein was dialysed (Spectra/Por® 4 membrane, molecular weight cut off 12-14 kDa) against 50 mM sodium phosphate buffer (pH 7.4), flash frozen in liquid nitrogen as aliquots and stored at -80°C.

Recombinant expression of B10-myc

Recombinant myc-tagged B10, whose amino acid sequence is analogous to the one expressed in *Drosophila* (SI Fig. 1C), was expressed from a custom synthesised gene (GeneArt) that was codon optimized for *E.coli* expression. The gene was cloned into the p416His vector and expressed in BL21(DE3) *E.coli* cells (Invitrogen). B10-myc protein was purified with an ÄKTA Explorer apparatus (GE Healthcare) and through a combination of nickel chelate (Nickel

Sepharose Fast Flow medium, GE Healthcare) and reversed phase chromatography (Source 15RPC medium, GE Healthcare).

Quantification of Aβ peptide, KW1 and B10 in head homogenates

WB was used also for the quantitative assessment of Aβ peptide concentrations and KW1/B10 concentration in fly head homogenates. The quantification was based on the comparison of the homogenate staining intensity with standard series of recombinantly expressed Aβ peptide or B10-myc antibody fragment. The standards for Aβ were prepared in increasing amounts from 0.01 - 1.0 ng. The investigated samples were prepared from heads of Aβ40, Aβ42 or Aβ42arc. The heads were homogenised in 15 µl PBS supplemented with 1 % SDS, sonicated for 8 min and spun down for 7 sec. The standard series and fly samples were analysed using SDS-PAGE and WB. To quantify the antibody domains B10-myc was used to create a standard from 0.5-5 ng. Heads were derived from B10 or KW1 expressing flies and analysed as described above. Densitometric quantifications of the detected bands in the WB were carried out with TotalLab 100 software and the molarities of Aβ or antibody fragments was calculated assuming a head volume of 65nl [1]. Reported peptide quantities represent medium ± standard error of mean (n=2-3).

Supporting References

1 Berg I, Thor S,Hammarström P (2009) Modeling Familial Amyloidotic Polyneuropathy (Transthyretin V30M) in Drosophila melanogaster. Neurodegenerative Dis 6: 127–13.

Supporting Figures and Figure Legends

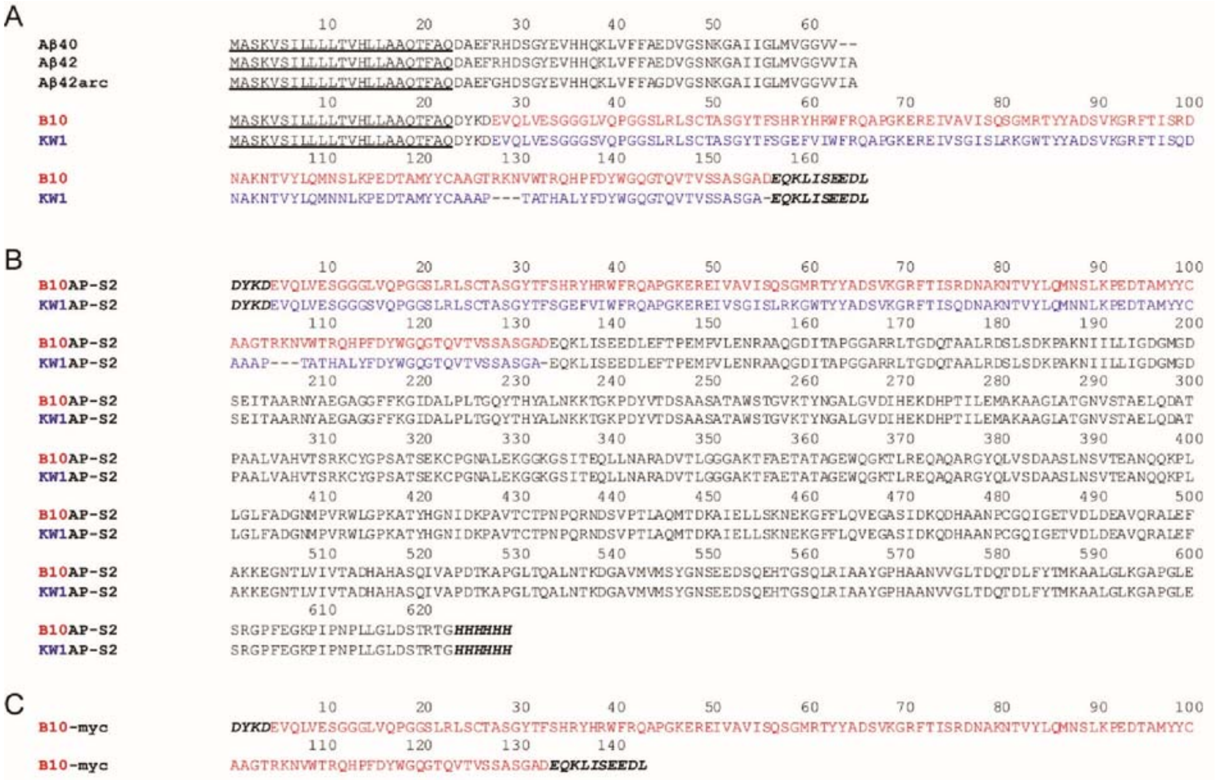

SI Figure 1.

Amino acid sequences of the expressed polypeptide chains.

Polypeptide chains expressed in *Drosophila* flies (**A**), in *Drosophila* S2 cells (**B**) and in *Escherichia coli* (**C**). Underlined is the secretion signal peptide, which is cleaved during protein biogenesis. Flag-tags, myc-tags and his-tags are marked in bold and italic. The coding sequence for B10 is highlighted in red and the coding sequence for KW1 is highlighted in blue.

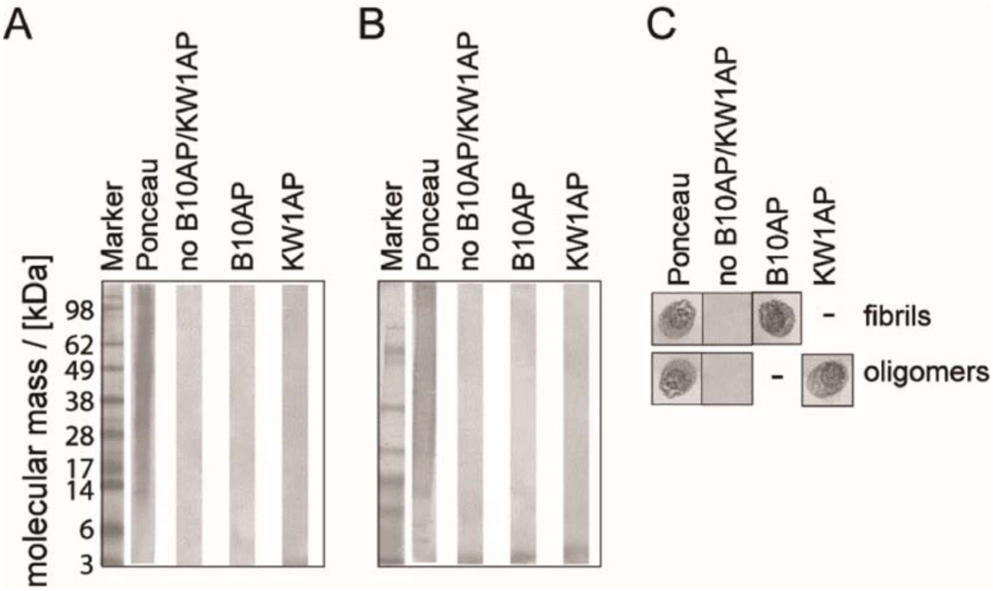

SI Figure 2.

B10AP and KW1AP do not cross-react with *Drosophila* proteins.

(**A-B**) WBs of WT *Drosophila* head homogenates separated out with denaturing SDS PAGE (**A**) or native PAGE (**B**) and probed with 4µg/ml B10AP or KW1AP in PBST (1 h, room temperature). The membrane was then washed three times for 5 min in PBST and developed with NBT/BCIP reagent. One lane was developed without B10AP or KW1AP to exclude endogenous alkaline phosphatase activity. The pre-stained marker SeeBlue® Plus2 (Invitrogen) was used in (**A**) to determine the size and in (**B**) as an additional transfer control. (**C**) B10AP and KW1AP activity controls with 20 µg Aβ(1-40) fibrils or oligomers spotted onto nitrocellulose membrane. Ponceau staining always served as a loading control.

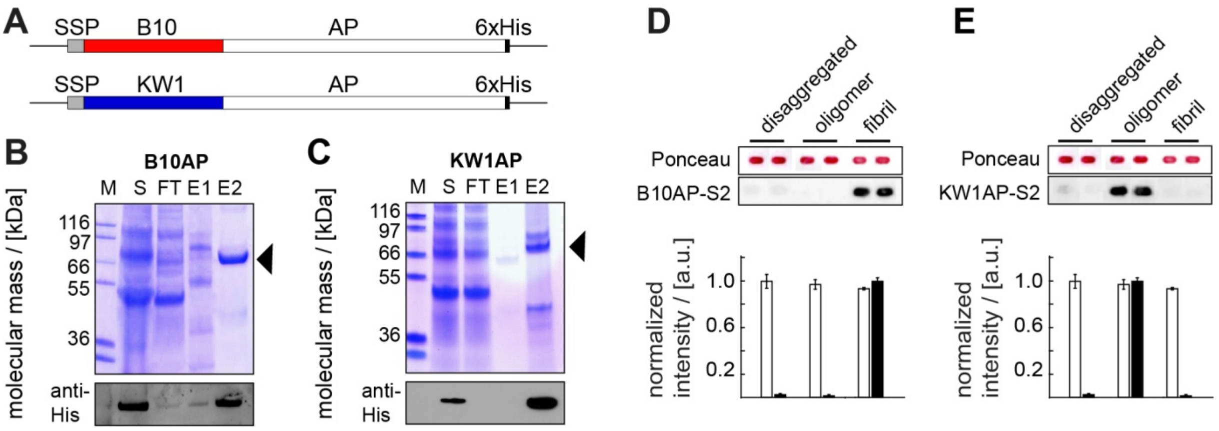

SI Figure 3.

***Drosophila* Schneider S2 cells are able to express functional KW1 and B10 antibody fragments.**

To determine whether the absence of any modulatory effect by B10 on Aβ-induced neurotoxicity could be attributed to a failure of *Drosophila* cells to synthesise and secrete functional B10 protein, *Drosophila* Schneider S2 cells were transformed with vectors encoding for B10 or KW1 antibody fragments in fusion with alkaline phosphatase (AP) to generate the two constructs B10AP-S2 and KW1AP-S2 (SI Fig. 1B). AP fusion enables direct visualization of binding in spot blots without the need of a secondary antibody. (**A**) Schematic representation of the used B10AP-S2 and KW1AP-S2 gene constructs. (**B, C**) Coomassie stained SDS PAGE (top) and WB (bottom, developed with anti-histidine antibody) to monitor the purification of B10AP-S2 (**B**) and KW1AP-S2 (**C**). Arrow heads indicate the band shown in the WB. Abbreviations: molecular weight marker (M), applied cell culture supernatant (S), flow through (FT) and elution steps E1 and E2. (**D, E**) Spot blots and densitometric quantifications (n = 2-3) of B10AP-S2 (**D,** black) and KW1AP-S2 binding (**E**, black) to Aβ(1-40) fibrils, oligomers or disaggregated peptide. Ponceau staining (white bar) serves as a loading control. B10AP-S2 shows specific binding to Aβ fibrils but not to oligomeric or disaggregated Aβ(1-40) peptide, whereas KW1AP-S2 solely binds oligomers.

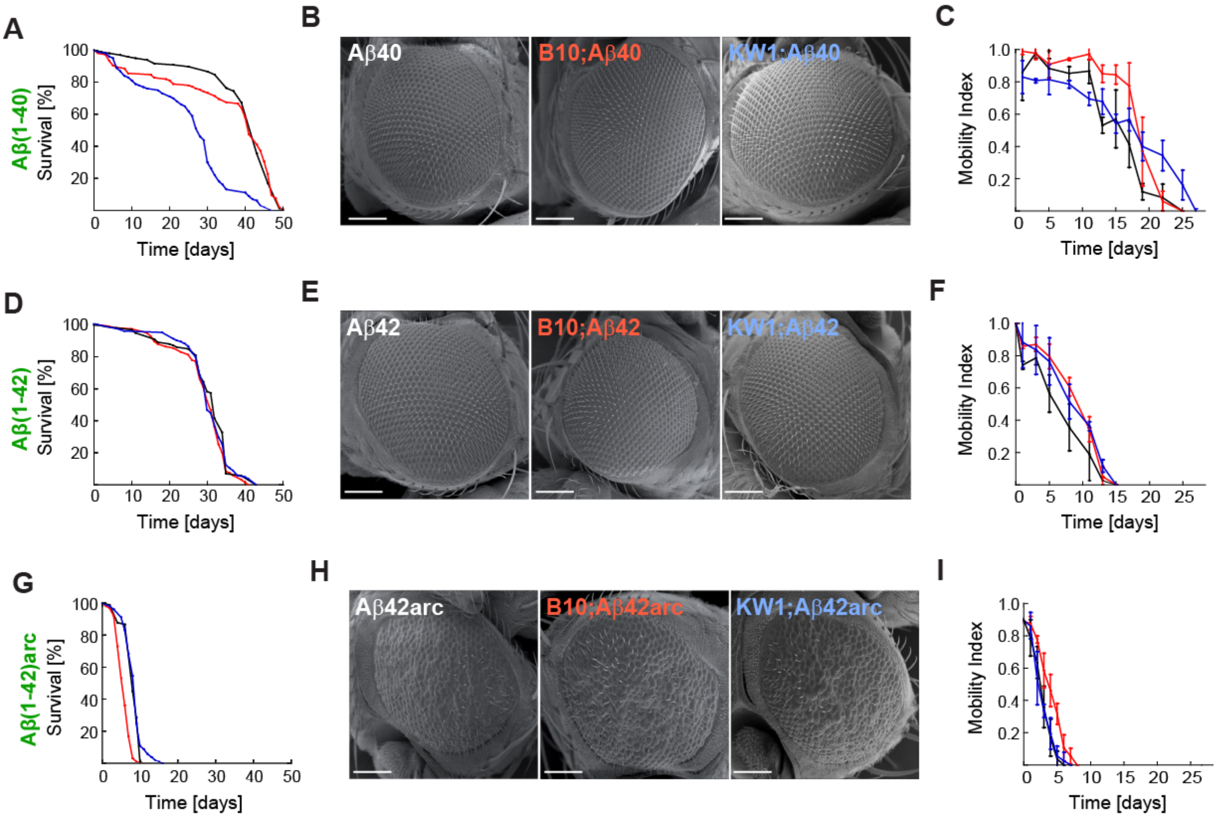

SI Figure 4.

Phenotypic effects of KW1 and B10 expression on Aβ-transgenic flies.

(**A-C**) flies expressing Aβ(1-40) peptide; (**D-F**) flies expressing Aβ(1-42) peptide; (**G-H**) flies expressing Aβ(1-42)arc peptide. Colour coding in all panels: white/black, no antibody fragment expressed; red, B10; blue, KW1. (**A,D,G**) Lifespan measurements of B10 and KW1 expressed with Aβ variants, (**B,E,H**) scanning electron microscopy images of the eye morphology (**C,F,I**) and negative geotaxis assay. Error bars show standard deviation from three independent experiments using 15 flies each.

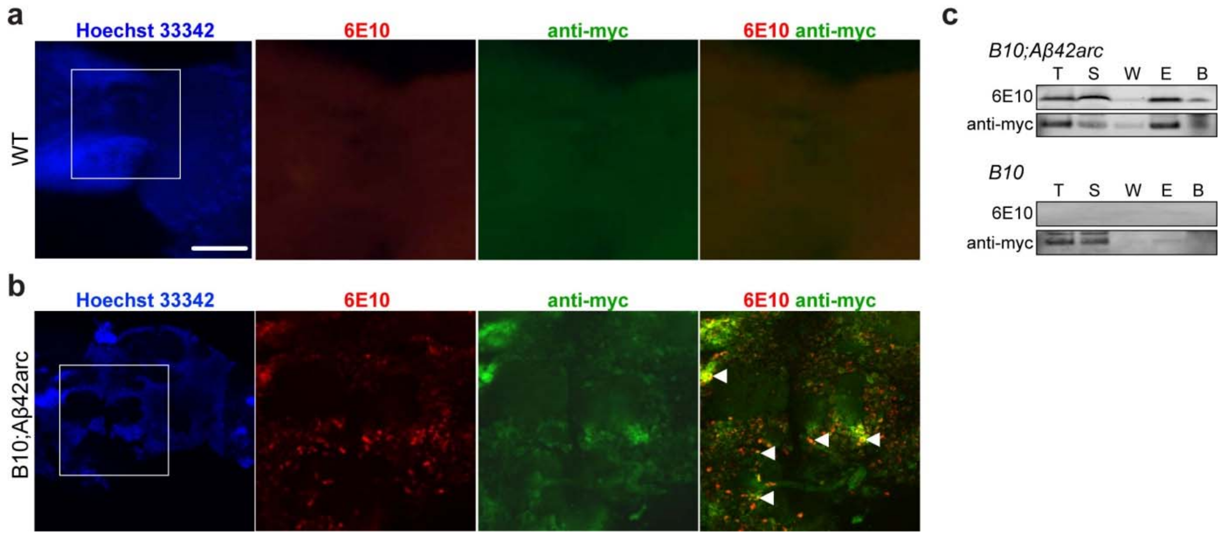

SI Figure 5.

B10 interacts with Aβ peptide in tissue samples.

(**A,B**) IFM images of adult brains from a 3-day old WT (**A**) or B10;Aβ42arc (**B**) flies. Left column (blue): Hoechst 33342 staining. White boxes indicate the regions enlarged in the following 3columns. Second column (red): Aβ-staining with 6E10 antibody. Third column (green): anti-myc antibody staining of myc-tagged B10. Right column: overlay of the anti-myc and 6E10 signals. Arrows indicate signal co-localisation, Hoechst 33342 staining visualises neuronal cell bodies. Scale bars represent 50 µm. (**C**) IP analysis of head homogenates from B10;Aβ42arc and B10 flies. Pull downs were performed against Aβ peptide and the resulting fractions were analysed with WB to detect Aβ (6E10) and myc-tagged B10 (anti-myc). Abbreviations: T, total sample before IP; S, supernatant after incubation with the beads; W, wash fraction; E, elution; B, beads after elution.

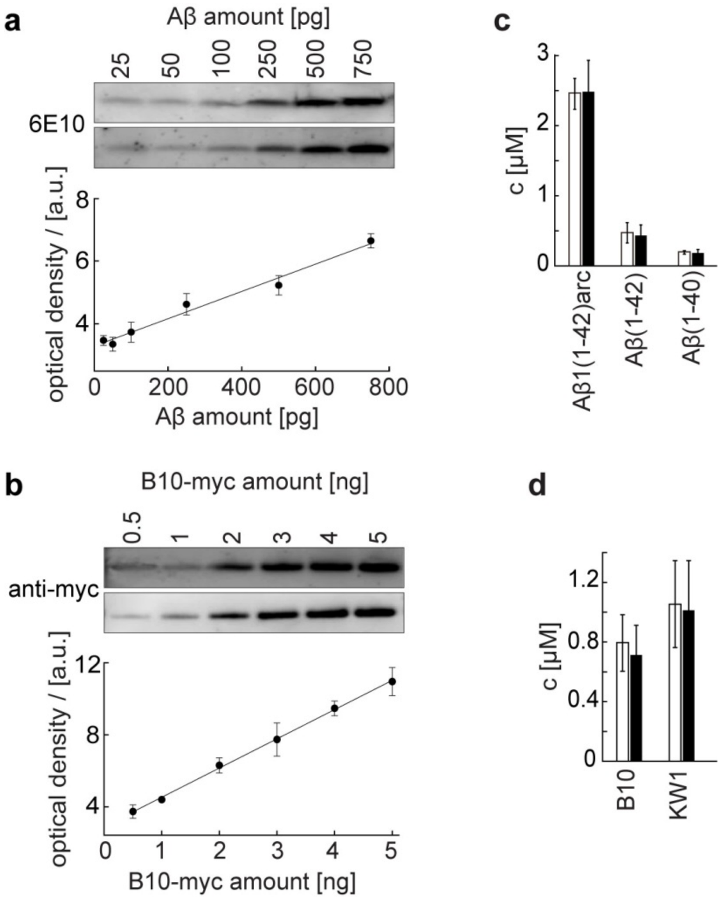

SI Figure 6.

Concentrations of Aβ, B10 and KW1 in fly head homogenates.

(**A-B**) Quantitative WB with Aβ (A) and B10-myc (B). Top: two representative WB lanes of the standard series. Bottom: densitometric quantifications fitted with a straight line. Error bars represent standard deviation (n=3). (**C**) Aβ concentration within the heads of Aβ40, Aβ42 and Aβ42arc flies after SDS (white bars) and urea (black bars) extraction. (**D**) B10 and KW1 concentrations within the heads of B10 and KW1 flies after SDS (white bars) and urea (black bars) extraction.
